# Terrestrial and lacustrine gastropods from the Priabonian (upper Eocene) of the Sultanate of Oman

**DOI:** 10.1007/s12542-015-0277-1

**Published:** 2015-10-29

**Authors:** Mathias Harzhauser, Thomas A. Neubauer, Dietrich Kadolsky, Martin Pickford, Hartmut Nordsieck

**Affiliations:** 1 Natural History Museum Vienna, Burgring 7, 1010 Vienna, Austria; 2 66 Heathhurst Road, Sanderstead, CR2 0BA Surrey, UK; 3 Sorbonne Universités-CR2P, MNHN, CNRS, UPMC-Paris VI, 8, rue Buffon, 75005 Paris, France; 4 Senckenberg Forschungsinstitut Frankfurt; Malakologie, Senckenberganlage 25, 60325 Frankfurt Am Main, Germany

**Keywords:** Mollusca, Arabian Peninsula, Biogeography, Paleogene, Palaeoecology, Gastropoda, Sultanat Oman, Biogeographie, Eozän, Paläoökologie

## Abstract

Terrestrial and aquatic gastropods from the upper Eocene (Priabonian) Zalumah Formation in the Salalah region of the Sultanate of Oman are described. The assemblages reflect the composition of the continental mollusc fauna of the Palaeogene of Arabia, which, at that time, formed parts of the southeastern Tethys coast. Several similarities with European faunas are observed at the family level, but are rarer at the genus level. These similarities point to an Eocene (Priabonian) rather than to a Rupelian age, although the latter correlation cannot be entirely excluded. At the species level, the Omani assemblages lack any relations to coeval faunas. This suggests the possible presence of a distinct biogeographic province during the Palaeogene or may simply reflect the extremely sparse non-marine fossil record of the Eocene in the Tethys region. The occurrence of the genera *Lanistes, Pila,* and *Gulella* along with some pomatiids, probably related to extant genera, suggests that the modern African–Arabian continental faunas can be partly traced back to Eocene times and reflect very old autochthonous developments. In contrast, the diverse Vidaliellidae went extinct, and the morphologically comparable Neogene Achatinidae may have occupied the equivalent niches in extant environments. *Carnevalea* Harzhauser and Neubauer nov. gen., *Arabiella* Kadolsky, Harzhauser and Neubauer nov. gen., *Pyrgulella* Harzhauser, Kadolsky and Neubauer nov. gen., *Salalahia* Kadolsky, Harzhauser and Neubauer nov. gen., *Omanitopsis* Harzhauser and Neubauer nov. gen., *Arabicolaria* Harzhauser and Neubauer nov. gen., *Pacaudiella* Harzhauser and Neubauer nov. gen., *Goniodomulus* Harzhauser and Neubauer nov. gen., *Eoquickia* Harzhauser and Neubauer nov. gen., *Omanillya* H. Nordsieck nov. gen. and *Omanifera* H. Nordsieck nov. gen. are introduced as new genera. *Pila neuberti* Harzhauser and Neubauer nov. sp., *Arabiella arabica* Kadolsky, Harzhauser and Neubauer nov. sp., *Pyrgulella parva* Harzhauser, Kadolsky and Neubauer nov. sp., *Salalahia thaytinitiensis* Kadolsky, Harzhauser and Neubauer nov. sp., *Omanitopsis vandammei* Harzhauser and Neubauer nov. sp., *Procyclotopsis eocenica* Harzhauser and Neubauer nov. sp., *Palaeocyclotus kuehschelmi* Harzhauser and Neubauer nov. sp., *Arabicolaria arabica* Harzhauser and Neubauer nov. sp., *Pacaudiella omanica* Harzhauser and Neubauer nov. sp., *Pacaudiella flammulata* Harzhauser and Neubauer nov. sp., *Goniodomulus solaniformis* Harzhauser and Neubauer nov. sp., *Cerastus hyznyi* Harzhauser and Neubauer nov. sp., *Omanillya lunellifera* H. Nordsieck nov. sp., *Omanillya costellata* H. Nordsieck nov. sp., and *Omanifera euclista* H. Nordsieck nov. sp. are described as new species.

## Kurzfassung

Terrestrische und aquatische Gastropoden aus der obereozänen (Priabonium) Zalumah-Formation in der Salalah-Region des Sultanats Oman werden beschrieben. Die Vergesellschaftungen reflektieren die Zusammensetzung der kontinentalen Molluskenfaunen des Paläogens Arabiens, das zu dieser Zeit die südöstliche Küste der Tethys formte. Ähnlichkeiten mit europäischen Faunen lassen sich auf Familien- und selten auch auf Gattungsniveau nachweisen. Diese Übereinstimmungen sprechen eher für ein eozänes Alter (Priabonium) als für eine Einstufung ins Rupelium, obwohl letzteres nicht völlig ausgeschlossen werden kann. Auf Artniveau fehlen Bezüge zu gleichalten Faunen. Das könnte auf eine diskrete paläogene Bioprovinz hinweisen oder einfach aus dem extrem dürftigen nicht-marinen Fossilbefund des tethyalen Eozäns resultieren. Das Vorkommen von Gattungen wie *Lanistes, Pila* und *Gulella*, gemeinsam mit einigen Pomatiiden, die mit rezenten Gattungen verwandt sein dürften, zeigt, dass die Wurzeln der modernen afrikanisch-arabischen kontinentalen Molluskenfauna zumindest teilweise bis ins Eozän zurückreichen. Im Gegensatz dazu starben die artenreichen Vidaliellidae aus, und die morphologisch ähnlichen Achatinidae dürften ihre ökologischen Nischen im Neogen übernommen haben. *Carnevalea* Harzhauser und Neubauer nov. gen., *Arabiella* Kadolsky, Harzhauser und Neubauer nov. gen., *Pyrgulella* Harzhauser, Kadolsky und Neubauer nov. gen., *Salalahia* Kadolsky, Harzhauser und Neubauer nov. gen., *Omanitopsis* Harzhauser und Neubauer nov. gen., *Arabicolaria* Harzhauser und Neubauer nov. gen., *Pacaudiella* Harzhauser und Neubauer nov. gen., *Goniodomulus* Harzhauser und Neubauer nov. gen., *Eoquickia* Harzhauser und Neubauer nov. gen., *Omanillya* H. Nordsieck nov. gen. und *Omanifera* H. Nordsieck nov. gen. Werden als neue Gattungen eingeführt. *Pila neuberti* Harzhauser und Neubauer nov. sp., *Arabiella arabica* Kadolsky, Harzhauser und Neubauer nov. sp., *Pyrgulella parva* Harzhauser, Kadolsky und Neubauer nov. sp., *Salalahia thaytinitiensis* Kadolsky, Harzhauser und Neubauer nov. sp., *Omanitopsis vandammei* Harzhauser und Neubauer nov. sp., *Procyclotopsis eocenica* Harzhauser und Neubauer nov. sp., *Palaeocyclotus kuehschelmi* Harzhauser und Neubauer nov. sp., *Arabicolaria arabica* Harzhauser und Neubauer nov. sp., *Pacaudiella omanica* Harzhauser und Neubauer nov. sp., *Pacaudiella flammulata* Harzhauser und Neubauer nov. sp., *Goniodomulus solaniformis* Harzhauser und Neubauer nov. sp., *Cerastus hyznyi* Harzhauser und Neubauer nov. sp., *Omanillya lunellifera* H. Nordsieck nov. sp., *Omanillya costellata* H. Nordsieck nov. sp. und *Omanifera euclista* H. Nordsieck nov. sp. werden als neue Arten beschrieben.

## Introduction

Fossils from Palaeogene strata of the Zalumah Formation cropping out in Dhofar Province, Sultanate of Oman, have been described in a number of publications (Thomas et al. 1988, 1989, 1991, 1992, 1999; Roger et al. 1993; Gheerbrant et al. 1993, 1995; Pickford and Thomas 1994; Neubert and Van Damme 2012; Pickford et al. 1994, 2014). This is the third paper devoted to the Eocene (or lowermost Oligocene) continental mollusc fauna of the Zalumah Formation in the Salalah region. Apart from some remarks in geological reports by Roger et al. (1989), Neubert and Van Damme (2012) were the first to describe a small gastropod assemblage from Haluf and Thaytiniti. These authors already recognised the assemblage as completely new and introduced several new species names for some of the better preserved specimens. Simultaneously, Pickford et al. (2014) focussed on the assemblages from Taqah in the Wadi Darbat and provided a first revision of the fauna, proposing several new combinations. Both teams focussed on the relations of the Omani taxa to modern Afro-Arabian genera and were limited in their identifications by the rather poor preservation of their material. An intense sampling campaign by the Franco-Omani Palaeontology Mission resulted in new, voluminous and much better preserved material from the same sections. Therefore, some of the previous determinations can be revised and new data on morphological features can be added to species described by Neubert and Van Damme (2012). Moreover, this newly collected material contains at least 20 species which were not present in the samples available to Neubert and Van Damme (2012) in their pioneer paper.

## Localities and geological setting

**Fig. 1:**
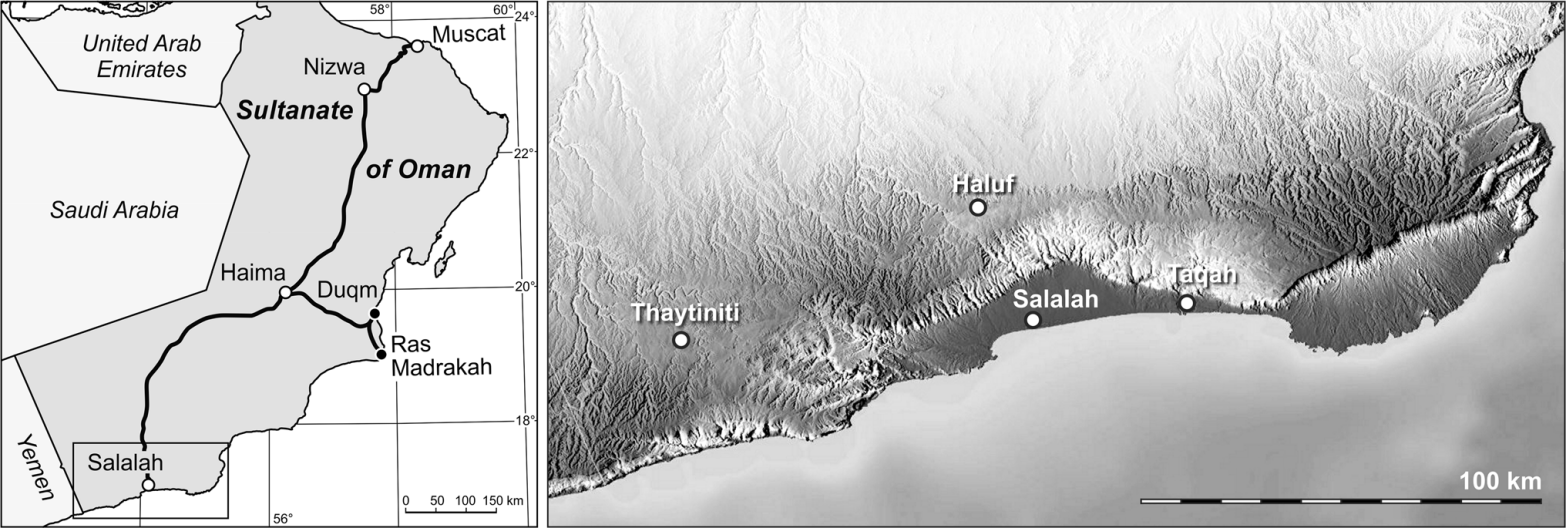
Geographic position of the investigated sections in the Dhofar region in the southwestern part of the Sultanate of Oman

Detailed descriptions of the geological setting are given by Roger et al. (1989, 1993) and Pickford et al. (2014), who also provide an overview on the papers dealing with the famous vertebrate faunas of the region. Sampling took place in January 2013 at the three localities Taqah (TQ), Thaytiniti (TN), and Haluf (HF) (Fig. 1), where fossiliferous, biomicritic white to beige freshwater limestones of the Zalumah Formation crop out close to sea level in the Wadi Darbat and at altitudes of over 700 m at Thaytiniti and the Haluf Graben. A detailed sedimentological description of the sections, however, has not been published so far.

The age of the Zalumah Formation is given as Priabonian to early Rupelian by Pickford et al. (2014), but unequivocal data are still lacking. The Eocene age estimate was based on the occurrence of a single charophyte species (Senut 1988). A better argument for the Eocene age is the occurrence of the Rupelian *Nummulites fichteli* in the base of the overlying Ashawq Formation (Senut 1988). Sea level rise during this period led to the accumulation of near-shore lacustrine facies at Wadi Darbat, Haluf, and Thaytiniti, all of which were, at the time, close to sea level as shown by the juxtaposition of strata yielding marine and continental faunas. The resulting freshwater deposits are dominated by carbonates of the Zalumah Formation rich in molluscs, but also with many vertebrate fossils in the overlying, more clastic Ashawq Formation. Subjacent marine beds are rich in marine fossils (Thomas et al. 1988, 1989, 1991, 1992, 1999; Gheerbrant et al. 1993, 1995; Pickford and Thomas 1994; Pickford et al. 1994, 2014). Post-Oligocene tectonic activity related to opening of the Red Sea and the Gulf of Aden led to uplift of the Dhofar Mountains, carrying the Thaytinini and Haluf mollusc assemblages with them (930 m asl at Thaytiniti, 750 m asl at Haluf), but left the Wadi Darbat occurrences close to sea level (25–40 m asl) (Pickford et al. 2014). Given the large geographic area and poor bio- and lithostratigraphy, it might be expected that the three investigated sections are not strictly coeval. This is also indicated by the quite different composition of the gastropod assemblages described herein.

Neubert and Van Damme (2012) published an article on freshwater and terrestrial molluscs from “Wadi Darbat” with the geographic coordinates 17.2514°N, 53.9826°E and 17.2586°N, 54.0060°E. This position corresponds to an outcrop in the Haluf Graben, 50 km NW of Wadi Darbat at an altitude of ca. 745 m. Wadi Darbat fossils collected by Pickford et al. (2014) are from outcrops ca. 25 m above sea level, close to Taqah, near the coast (17°02′43.5″N, 54°26′51.1″E). Thaytiniti located at 16°54′52.5″N, 53°25′47.9″E at an altitude of 920 m is the type locality of *Lanistes thaytinitiensis, Tropidophora praecursor* and *Limicolaria omanensis* of Neubert and Van Damme (2012). See Table 1 for exact positions and altitudes of the samples investigated herein.

**Table 1:** Geographic positions and and altitudes of the samples, with sample code numbers

Locality	Latitude	Longitude	Altitude (m)	Code
Taqah 2	17°02′43.5″N	54°26′51.1″E	25	TQ2
Taqah 3	17°02′44.7″N	54°26′43.4″E	29	TQ3
Thaytiniti 3A	16°57′28.0″N	53°17′03.1″E	926	TN3a
Thaytiniti 3B	16°57′33.5″N	53°17′07.1″E	921	TN3b
Thaytiniti 3C	16°57′29.9″N	53°17′04.1″E	924	TN3c
Thaytiniti 8	16°57′23.1″N	53°15′03.4″E	907	TN8
Thaytiniti 9	16°59′28.0″N	53°13′43.6″E	869	TN9
Thaytiniti 12	16°54′47.0″N	53°25′43.9″E	915	TN12
Thaytiniti 15B	16°55′48.5″N	53°25′19.7″E	929	TN15b
Thaytiniti 15C	16°55′49.8″N	53°25′19.5″E	928	TN15c
Thaytiniti 17B	16°57′37.2″N	53°21′58.6″E	921	TN17b
Haluf 1a	17°15′04.0″N	53°57′44.0″E	749	HF1a
Haluf 1d	17°15′06.4″N	53°57′43.6″E	746	HF1d
Haluf 3B	17°15′18.6″N	53°58′29.5″E	750	HF3b
Haluf 4B	17°15′28.4″N	53°58′46.7″E	774	HF4b
Haluf 4C	17°56′26.7″N	53°58′46.9″E	764	HF4c

## Material

All specimens were exported under a license approved by the Directorate General of Minerals of the Sultanate of Oman, Muscat. All type specimens are stored at the Oman Natural History Museum (ONHM) in Muscat, all additional material is stored in the Muséum national d’Histoire naturelle (MNHN) in Paris.

## Systematic palaeontology

The systematic arrangement of higher taxa largely follows the proposals of Bouchet and Rocroi (2005) and Wade et al. (2006). While we generally use the descriptive terminology of Cox (1960a) for gastropod shells, his nomenclature for describing the direction and shape of growth lines relative to the axis is incomplete: Cox named five shapes (orthocline, prosocline, opisthocline, prosocyrt, opisthocyrt), although theoretically nine are possible: three main directions: parallel to the axis (=orthocline), running forward adapically (=prosocline), running backward adapically (=opisthocline). Each of them can be straight, convex, or concave, the latter two as seen in the direction of shell growth. Thus, Cox’s five terms become the following: opisthocyrt = orthocline concave, prosocline = prosocline convex, orthocline = orthocline straight, opisthocline = opisthocline concave, and prosocyrt = orthocline convex. Terminology of pomatiid opercula follows Wilmsmeier and Neubert (2012).

Class Gastropoda Cuvier, 1795

Subclass Caenogastropoda Cox, 1960b

Unassigned order

Superfamily Ampullarioidea Gray, 1824

Family Ampullariidae Gray, 1824

**Genus** ***Pila*** **Röding**, 1798

*Type species*: *Helix ampullacea* Linnaeus, 1758; subsequent designation by Dall (1904). Recent, Southeast Asia.

***Pila*** ***neuberti*** **Harzhauser and Neubauer nov. sp.**

Figure 2c–e

**Fig. 2:**
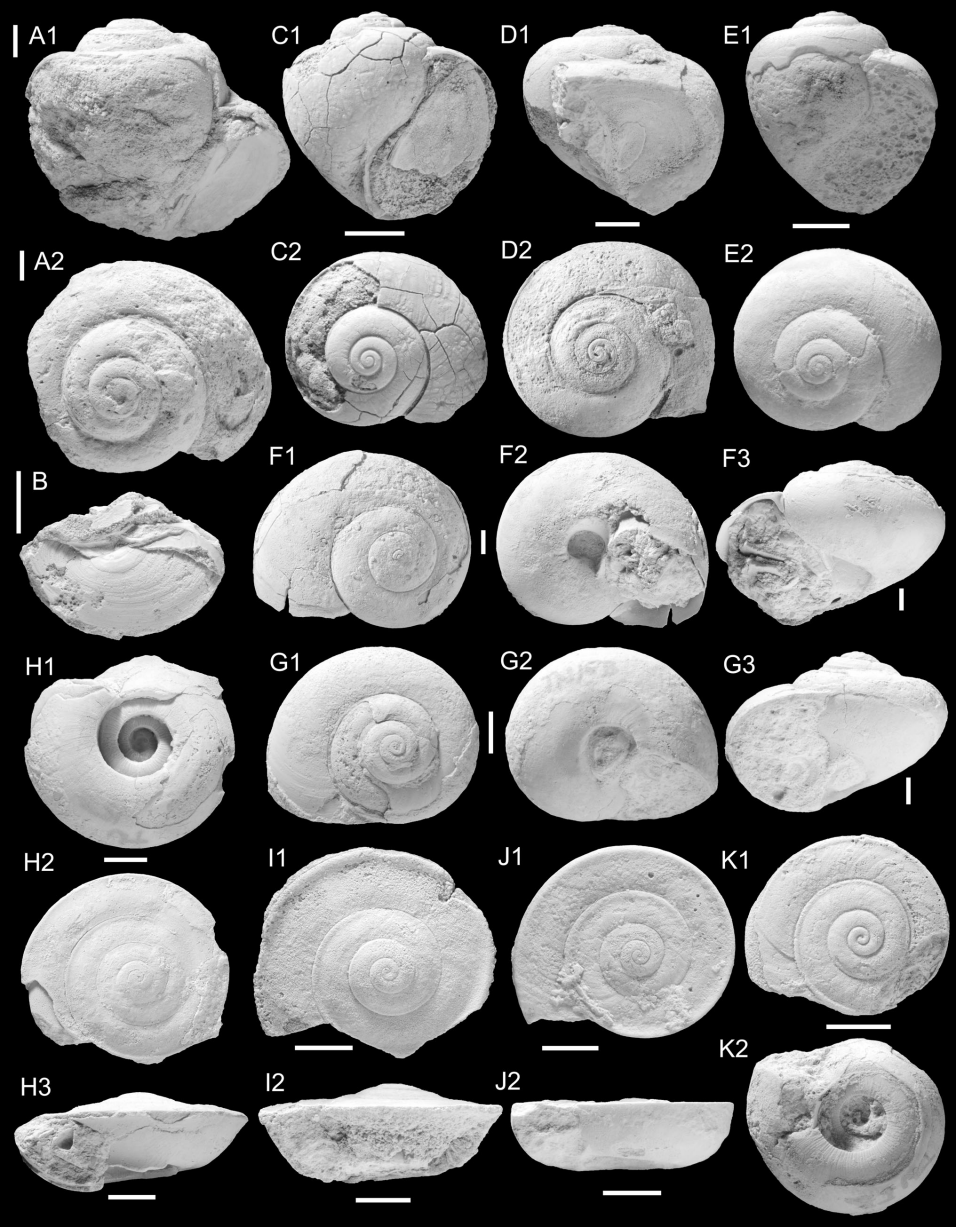
Ampullariidae. **a**, **b** *Pila* sp. **b** is operculum of **a**, TN3b; ONHM/TN/0004. **c**–**e** *Pila* *neuberti* Harzhauser and Neubauer nov. sp. **c** Holotype, TN3c; ONHM/TN/0001. **d** Paratype, TN3b; ONHM/TN/0002, **e** paratype. TN9; ONHM/TN/0003. **f**–**g** *Lanistes tricarinatus* Neubert and Van Damme, 2012. **f** TN3a; ONHM/TN/0005, **g** TN15b; ONHM/TN/0006. **h**–**k** *Carnevalea thaytinitiensis* (Neubert and Van Damme, 2012). **h** TN15b; ONHM/TN/0009. **i** TN8; ONHM/TN/0007. **j** TN8; ONHM/TN/0008. **k** TN15b; ONHM/TN/0010. *Scale bar* 5 mm

2014 *Pila* sp. 2.—Pickford et al.: 96.

*Holotype*: Fig. 2c, ONHM/TN/0001, height: 17.5 mm, diameter: 17.5 mm (TN3c).

*Paratype*: Fig. 2d, ONHM/TN/0002, height: 23 mm, diameter: 25 mm (TN3b).

*Paratype*: Fig. 2e, ONHM/TN/0003, height: 19 mm, diameter: 18 mm (TN9).

*Additional material*: 15 (TN3a), 6 (TN3c), 1 (TN9), 8 (TN12).

*Measurements*: Largest specimen: height: 26 mm, diameter: 31 mm.

*Stratum typicum*: Biomicritic limestones of the Zalumah Formation.

*Type locality*: Thaytiniti, near Salalah, Sultanate of Oman.

*Age*: Priabonian (or early Rupelian).

*Name*: In honour of Eike Neubert, Zoologist at the Natural History Museum Bern and pioneer of the study of Eocene continental gastropods from the Sultanate of Oman.

*Description*: Small to medium-sized *Pila* consisting of ca. four whorls, displaying slightly allometric growth. Low spire ranging from nearly flat to slightly elevated, with evenly convex whorls increasing slowly in diameter; suture deeply incised. Subadult shells lacking the last whorl are weakly convex along the periphery and contract rapidly into the umbilical side of the shell, producing a somewhat reverse-conical outline. Last whorl strongly widening and convex, resulting in a shell broader than high. Shell surface smooth except for delicate, prosocline growth lines. Aperture semi-circular with slightly rounded posterior tip and very narrow, reflected columellar lip, demarcating a narrow and deep umbilicus. Operculum thin with faint growth lines and deep concavity around the nucleus; parietal margin only weakly thickened.

*Remarks*: Apart from the constantly smaller size and the lower spire, this species differs from the co-occurring *Pila* sp. also in its operculum, which is thinner, has weaker growth lines and a deeper concavity around the nucleus. Among the extant African *Pila* species, *P. cecillei* (Philippi, 1848) is somewhat reminiscent of the Eocene species concerning size and outline, but it has a higher spire, and the aperture is attached in a lower position. The reverse-conical shell outline is also typical for the Asian *P. ampullacea* (Linnaeus, 1758) (Ng et al. 2014), which is larger and has a more globular spire.

*Distribution*: Only known from Thaytiniti.

***Pila*** **sp.**

Figure 2a–b

2014 *Pila ovata*.—Pickford et al.: 96 (non *Ampullaria ovata* Olivier, 1804).

*Material*: 1 specimen from TN3b with in situ operculum (Fig. 2a–b; ONHM/TN/0004) and 2 opercula, which are tentatively assigned to this species based on their large size (TN3c, TN9).

*Measurements*: height: 40 mm, diameter: 31 mm.

*Description*: Medium-sized sub-globose shell with elevated, slightly gradate spire and deeply impressed suture. Teleoconch whorls strongly convex with narrow sutural shelf; aperture and base largely destroyed. Solid operculum, with concave outer side, delicate, concentric growth lines and a knob-like nucleus at the thickened parietal margin.

*Remarks*: This species was listed as *Pila ovata* by Pickford et al. (2014), to which it is superficially similar. The extant African species, however, is much larger. Schultheiß et al. (2009) proposed that *P. ovata* did not appear before the Miocene based on molecular clock analyses, which accords with the oldest fossil record from the Miocene of Kenya (Newton 1914; Kat 1987). *Pila* sp. from the Priabonian (or early Rupelian) of the Haluf area, described by Neubert and Van Damme (2012), is larger, has a globular shell and a shorter spire. A separation from *Pila colchesteri* Cox, 1933 from the Palaeogene Hudi Chert Formation in Sudan is not easy due to the poor preservation but *Pila colchesteri* is generally larger and the position of the aperture is higher, resulting in a more globular outline. A closer relation with *Afropomus* Pilsbry and Bequaert, 1927, which is a basal taxon in this family (Jørgensen et al. 2008), is unlikely as *Afropomus* has a thin operculum without alcareous layer (Pilsbry and Bequaert 1927).

*Distribution*: Only known from Thaytiniti.

**Genus** ***Lanistes*** **Montfort,** 1810

*Type species:* *Lanistes olivierii* Montfort, 1810 [=*Lanistes boltenianus* (Röding, 1798) = syn. *Lanistes carinatus* (Olivier, 1804)]; original designation. Recent, Egypt (see Lee 2013 for the complex nomenclatorial history of this species).

***Lanistes tricarinatus*** **Neubert and Van Damme**, 2012

Figure 2f–g

* 2012 *Lanistes tricarinatus* Neubert and Van Damme: 6, Figs. 5, 6.

2014 *Lanistes tricarinatus*.—Pickford et al.: 95, 96.

*Material*: 1 (TN3a, Fig. 2f; ONHM/TN/0005), 30 (TN8), 9 (TN15b, Fig. 2g; ONHM/TN/0006), 2 (HF4b).

*Measurements*: largest specimen: height: 39 mm, diameter: 53 mm.

*Remarks*: The eponymous sculpture on the second and third teleoconch whorl is rarely preserved (or developed) in the new material. This species belongs to an African species group that appears at least during the early Eocene. One of the oldest representatives is *Lanistes grabhami* Cox, 1933 from the lower Eocene of the Republic of the Sudan. This species differs from other Palaeogene species mainly by its slowly widening whorls (see Gautier, 1973). A slightly younger species is *Lanistes antiquus* Blanckenhorn, 1901 from the Lutetian of Egypt (Bellardi 1855; Mayer-Eymar 1901; Blanckenhorn 1901; Newton 1912). Blanckenhorn (1901) emphasised the presence of a weak median angulation of the last whorl as a diagnostic feature. Because such angulated specimens also appear in populations of the otherwise evenly rounded recent *L. carinatus*, the diagnostic value of this feature might be questioned. Neubert and Van Damme (2012) based the separation of their *L. tricarinatus* from *L. antiquus* only on the alleged smaller size of the latter, obviously overlooking the paper by Newton (1912), which shows a huge *L. antiquus* of 85 mm diameter from the Lutetian of Fayum (Egypt). This specimen is still present in the collections of the Natural History Museum in London (NHMUK G.24448) and shows an internal cast with evenly rounded last whorl. A separation from *L. tricarinatus* may only be based on its higher spire. Similarly problematic is the separation of *L. tricarinatus* (and *L. antiquus*) from the younger *Lanistes bartoninus* Blanckenhorn, 1901, from the middle Eocene of Egypt. This species was never illustrated and Blanckenhorn (1901) based it mainly on the evenly rounded last whorl (in contrast to *L. antiquus*). Thus, the status of all these species remains unclear. In conclusion, the occurrence of typical *Lanistes* in the Omani sections indicate a faunistic relation to the Egyptian Eocene, but the poor preservation of the Egyptian specimens makes comparisons difficult.

*Distribution*: Known from Thaytiniti (this paper) and from the Haluf area (Neubert and Van Damme 2012).

**Genus** ***Carnevalea*** **Harzhauser and Neubauer nov. gen.**

*Type species*: *Lanistes thaytinitiensis* Neubert and Van Damme, 2012. Eocene, Priabonian (or early Rupelian), Sultanate of Oman.

*Diagnosis*: Medium-sized, hypertrophic, discoidal, saucer-shaped shell with sharp keel, comprising about five whorls (including protoconch); nearly flat spire apart from the slightly pointed apex. Spire whorl weakly convex passing into a shallow concavity towards the periphery. Protoconch not protruding from early spire whorls (separation from teleoconch unclear). Weak sculpture formed by growth lines. Wide perspective umbilicus delimited by sharp, overhanging carina.

*Included species*: Only the type species is known so far.

*Name*: In honour of Giorgio Carnevale, palaeontologist at the Dipartimento di Scienze della Terra, Università degli Studi di Torino.

*Remarks*: When introducing *Lanistes thaytinitiensis*, Neubert and Van Damme (2012) emphasised that this species is the only discoid *Lanistes* known. In our opinion, the saucer-shaped shell outline, the sharp carina and *Architectonica*-like perspective and stepped umbilicus (instead of funnel-shaped) exclude a placement in *Lanistes* s.s. as defined by Wenz (1939) and Brown (1994). Moreover, *Lanistes* is already represented during the Eocene by species typical of the genus, such as *L. antiquus* Blanckenhorn, 1901, *L. tricarinatus* Neubert and Van Damme, 2012 and others (see Wenz 1928). Species of the *Lanistes*-related Eocene African-Arabian *Pseudoceratodes* Wenz, 1928, such as *P. mammuth* (Blanckenhorn, 1901) and *P. cairensis* (Abbass, 1962), differ clearly from both *Lanistes* and *Carnevalea* in their planorbid shape, which might suggest a closer relation to the extant *Marisa* Gray, 1824. Nevertheless, the sinistral shell supports a placement within the Ampullariidae.

***Carnevalea thaytinitiensis*** **(Neubert and Van Damme,** 2012)

Figure 2h–k

*2012 *Lanistes thaytinitiensis* Neubert and Van Damme: 9, Fig. 7.

2014 *Lanistes thaytinitiensis*.—Pickford et al.: 95, 96.

2014 *Lanistes* sp. 3.—Pickford et al.: 96.

*Material*: 179 (TN8, Fig. 2i, j ONHM/TN/0007, ONHM/TN/0008), 5 (TN15b, Fig. 2h, ONHM/TN/0009; 2k, ONHM/TN/0010).

*Measurements*: Largest specimen: height: 8 mm, diameter: 28 mm.

*Remarks*: This species was described by Neubert and Van Damme (2012) based on only a few poorly preserved specimens. The characteristic shape leaves little doubt that the new specimens are conspecific with *L. thaytinitiensis*. The much richer material now available from Thaytiniti, reveals limited variability in this species. Only the weak convexity in the middle of the spire whorls and the adjoining concavity are slightly variable and thus result in a more or less accentuated protrusion of the peripheral keel from the spire. In addition, the spire height is variable and ranges from nearly flat to low conical.

*Distribution*: Only known from Thaytiniti.

Order Littorinimorpha Golikov and Starobogatov, 1975

Superfamily Truncatelloidea Gray, 1840

?Family Hydrobiidae Stimpson, 1865

**Genus** ***Salalahia*** **Kadolsky, Harzhauser and Neubauer nov. gen**.

*Type species*: *Salalahia thaytinitiensis* nov. gen. nov. sp.; Eocene, Priabonian (or early Rupelian), Sultanate of Oman.

*Diagnosis*: Shell of ovate-conical shape with a truncated apex, up to 6 mm high, anomphalous; whorls weakly to moderately convex, last whorl rounded at the periphery. Protoconch with a 0.16 mm wide nucleus; adult shells are decollated, and the apex is closed with a secondary shell. Middle teleoconch whorls with prominent, widely spaced ribs, which tend to disappear on the last half whorl and which terminate at the periphery of the last whorl. Last half whorl strongly to weakly pulled-in, and immediately before the peristome appearing to expand due to the presence of a strong terminal varix. Palatal margin weakly opisthocline-concave; abapical apertural margin rounded; peristome thickened all round, with the columellar and parietal margin forming a single arc. A pseudumbilical chink is formed by the raised columellar margin.

*Name*: After the Omani city Salalah.

*Included species:* Only the type species is known so far.

*Remarks*: *Salalahia* is most similar, and probably related, to *Nystia* Tournouër, 1869 (as restricted by Kadolsky 1993), which is known only from the lower Rupelian of western Europe, particularly to *N. pseudoplicata* Glibert and de Heinzelin, 1954. Similar features are the size range, shell shape, the size of the nucleus, the extent of decollation and the shape of the secondary shell, the presence of ribs of similar size and profile outline in apical view (reduced or absent in some *Nystia* species), which also terminate at the periphery, the shape of the terminal varix and the pulled-in last half whorl. Distinguishing characters of *Nystia* are: the whorls are slightly more convex with correspondingly deep sutures, the umbilicus is usually open, and the peristome shows a tendency to flare adapically much stronger, and also to form a shallow sinus there, resulting in the long axis of the oval aperture being more oblique than in *Salalahia*; the palatal margin is more strongly opisthocline-concave. The species of *Nystia* occur in coastal waters and inland waters with indications of increased salinity, but not in clearly freshwater faunal associations.

It is unclear whether the similarities between *Salalahia* and *Nystia* are due to convergence or represent a true relationship. The latter cannot be ruled out as *Nystia* is known from the northwestern Tethys margin (southern France), and faunas of late Eocene to early Oligocene age in the intervening region are very incompletely known.

The similarity with the hydrobioid genus *Prososthenia* Neumayr, 1869, with which Pickford (2014) compared the type species, is thought to be due to convergence, as this genus occurs in brackish associations of the Paratethys and proto-Mediterranean realm in the middle Miocene to Pliocene. Several *Prososthenia* species were discussed recently by Neubauer et al. (2011, 2013a, b) including its middle Miocene type species *Prososthenia schwartzi* Neumayr, 1869. Although *Prososthenia* species are also characterized by a highly variable intraspecific variability in sculpture and despite some parallels in aperture morphology, a close relation to *Salalahia* can be excluded. *Prososthenia* has a narrower and higher last whorl, its aperture is thicker and often detached from the base, resulting in a much wider pseudumbilical chink; it does not decollate and it lacks a terminal varix.

***Salalahia thaytinitiensis*** **Kadolsky, Harzhauser and Neubauer nov. gen. nov. sp.**

Figure 3a–d

**Fig. 3:**
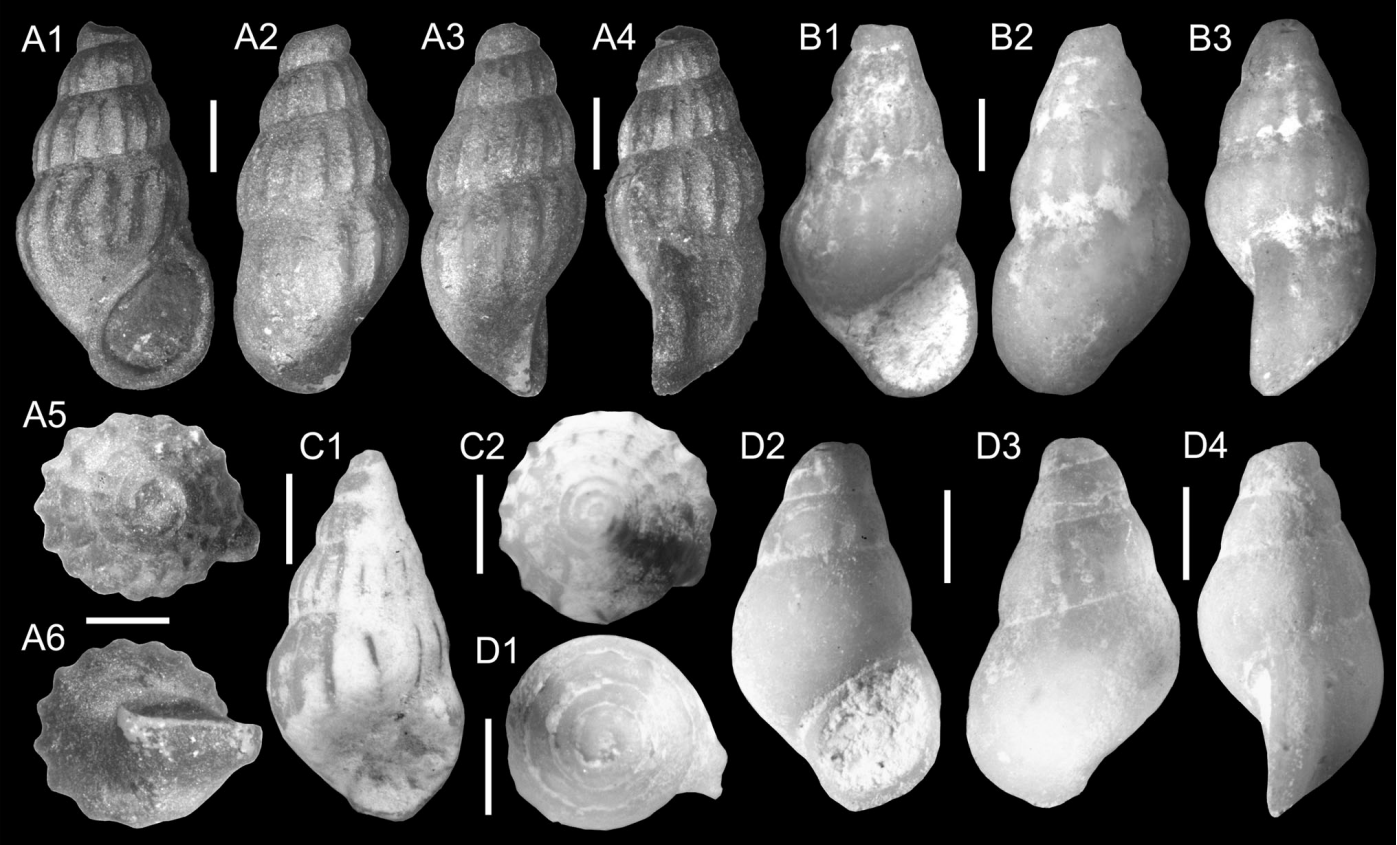
Hydrobiidae. **a**–**d** *Salalahia thaytinitiensis* Kadolsky, Harzhauser and Neubauer nov. sp. **a** Holotype, TN3a; ONHM/TN/0011. **b** TN3a; ONHM/TN/0012. **c** TN3a; ONHM/TN/0013. **d** Paratype, TN3a; ONHM/TN/0014. *Scale bar* 1 mm

2014 cf. *Prososthenia*. – Pickford et al.: 96.

*Holotype*: Fig. 3a, ONHM/TN/0011, height: 5.16 mm, diameter: 2.7 mm (TN3a).

*Paratypes*: Fig. 3b, ONHM/TN/0012, height: 5.0 mm, diameter: 2.6 mm (TN3a).

Figure 3c, ONHM/TN/0013, height: 4.12 mm, diameter: 2.2 mm (TN3a).

Figure 3d, ONHM/TN/0014, height: 3.94 mm, diameter: 2.25 mm (TN3a).

*Additional paratypes:* ca. 710 (TN3a), 15 (TN3c), 20 (TH12).

*Stratum typicum*: biomicritic limestones of the Zalumah Formation.

*Type locality*: Thaytiniti, near Salalah, Sultanate of Oman.

*Age*: Priabonian (or early Rupelian).

*Name*: After the Thaytiniti area.

*Diagnosis*: as for the genus (sole species).

*Remarks*: A single specimen out of ca. 710 from sample TN3a had not decollated and thus allowed us to ascertain the size of the nucleus and of the earliest whorls (Fig. 3c). Because of recrystallization, finer surface details such as the sculpture and boundary protoconch/teleoconch were not observable. The earliest ribbing was observed at a shell width of 0.94 mm at 2.7 whorls; it could have begun earlier in an area covered by rock matrix. In the same specimen the rock matrix adhering to the shell appears to have prevented abrasion of the ribs which have sharper crests than in many other specimens without rock matrix.

This species is very abundant in sample TN3a and reveals an enormous variability concerning shape and sculpture. Both the size of mature shells and their spiral angle (and hence the height/width ratio) are quite variable. The ribbing is in the majority strong except for the last half-whorl, but can become obsolete and even completely absent (ca. 5 % of sample TN3a). The disappearance of the ribs does not affect the development of the terminal varix which is always present. The convexity of the whorls varies somewhat, but this is influenced by the ribbing which makes the outline of the whorls appear more convex than it would otherwise be.

*Distribution*: Only known from Thaytiniti.

**Genus** ***Arabiella*** **Kadolsky, Harzhauser and Neubauer nov. gen.**

*Type species:* *Arabiella* *arabica* nov. gen. nov. sp.; Eocene, Priabonian (or early Rupelian), Sultanate of Oman.

*Diagnosis*: Shell of conical shape, up to 7 mm high, anomphalous, thick-walled; whorls weakly convex, sutures not appressed; last whorl weakly subangulate. Protoconch with relatively large nucleus (diameter ca. 0.16 mm), its sculpture and number of whorls not observable. Teleoconch with growth lines only, these prosocline and weakly concave. Aperture pear-shaped, with a long and straight columellar margin, well developed parietal callus and a broad and shallow sinus abapically. A pseudumbilicus is formed by the raised columellar margin.

*Included species*: The type species only.

*Remarks*. The habitus and size of *Arabiella* resemble those of *Lutetiella* Kadolsky, 2015 from the Lutetian of western Europe, as well as many Assimineidae. The distinguishing traits are listed in Table 2.

**Table 2:** Distinguishing traits in *Lutetiella, Arabiella* (both family Hydrobiidae?) and Assimineidae

	*Lutetiella*	*Arabiella*	Assimineidae
Nucleus	55–90 µm	160 µm	50–80 or 120–200 µm
Protoconch 1	Sculpture of confluent wrinkles; sometimes irregular short spiral crests	Details not observable	Very small tubercles, more or less connected by smaller wrinkles
Protoconch 2	With variable, sometimes coarse growth lines and broad, rounded spiral cords; number and position variable, often absent	Absent	Absent or present; when present, with numerous regular spiral threads
Sculpture	Growth lines of first and second order; fine spiral striae	Growth lines	Growth lines; some species with vestiges of spiral furrows, in particular subsuturally
Last whorl growth	Varies from regular growth to being pulled in under preceding whorl	Last quarter whorl expanded in width	Regular, neither expanding nor contracting
Growth lines	Prosocline weakly concave	Prosocline weakly concave	Prosocline strongly to weakly concave
Abapical apertural sinus	Absent	Broad and shallow	Absent
Umbilicus	Narrow to closed; pseudumbilicus present	Closed; pseudumbilicus present	Closed or open

Data from Brandt (1974), Fretter and Graham (1978), Fukuda and Ponder (2003), Janssen (2007), Kadolsky (2015), Kowalke (1998), van Aartsen (2008) and new data

From this, it is evident that the main distinguishing trait of *Arabiella* is the shallow abapical apertural sinus. The tentative family attribution to Hydrobiidae is the default option, with the caveats discussed, interalia, by Kadolsky (2015).

The diameter of the nucleus seems to be correlated with the presence or absence of a protoconch 2. The latter is recognizable by its sculpture which differs from that of protoconch 1 and the teleoconch. It is usually assumed that protoconch 1 is formed inside the egg, and protoconch 2 in the larval stage which may occur in the egg or may be truly planktic. A planktotrophic veliger larval stage is indeed known for *Assiminea grayana* Fleming, 1828, which has a very small nucleus and a protoconch 2 (Fretter and Graham 1978). The large protoconch size of *Arabiella* thus implies a direct development.

***Arabiella*** ***arabica*** **Kadolsky, Harzhauser and Neubauer nov. gen. nov. sp.**

Figure 4a–d

**Fig. 4:**
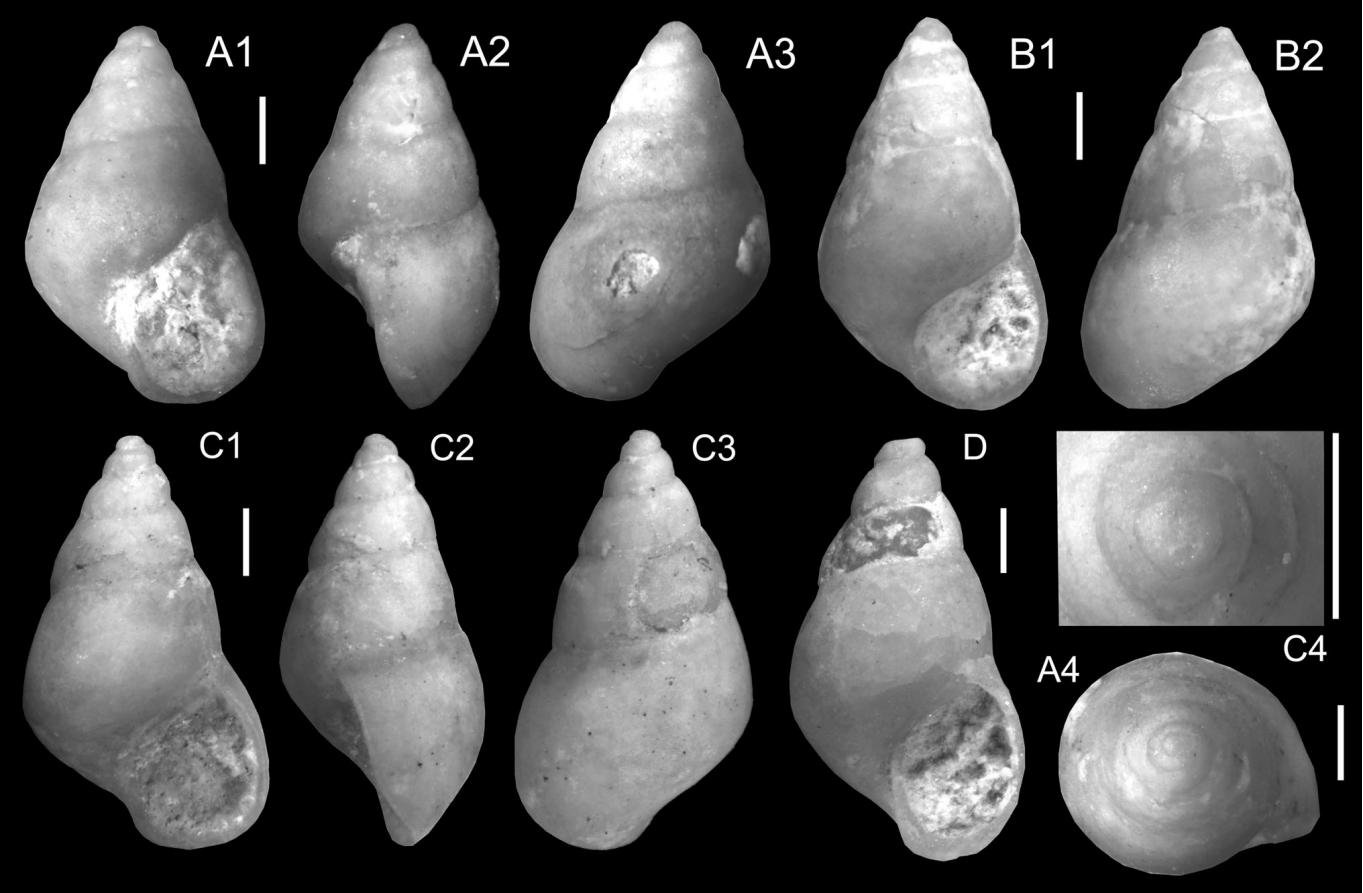
Hydrobiidae. **a**–**d** *Arabiella arabica* Kadolsky, Harzhauser and Neubauer nov. sp. **a** Holotype, TN17b; ONHM/TN/0015. **b** Paratype, TN17b; ONHM/TN/0016. **c** HF1d; ONHM/HF/0001. **d** HF1d; ONHM/HF/0002. *Scale bar* 1 mm

*Holotype*: Fig. 4a, TN17b, ONHM/TN/0015, number of whorls 5.0, height: 5.84 mm, width: 3.68 mm, apertural height: 2.76 mm, apertural width: 2.36 mm, height of last whorl: 4.2 mm, width of nucleus: 150 µm.

*Paratype*: Fig. 4d, TN17b, ONHM/TN/0016, number of whorls 4.9, height: 6.17 mm, width: 3.58 mm, apertural height: 2.67 mm, apertural width: 2.00 mm, height of last whorl: 4.25 mm, width of nucleus: 140 µm.

*Additional material*: Fig. 4b, HF1d, ONHM/HF/0001, number of whorls 5.3, height: 6.33 mm, width: 3.75 mm, apertural height: 3.00 mm, apertural width: 2.17 mm, height of last whorl: 4.33 mm, width of nucleus: 160 µm. Figure 4c, HF1d, ONHM/HF/0002, height: 6.83 mm, width: 3.17 mm, apertural height: 3.17 mm, apertural width: 2.17 mm, height of last whorl: 4.92 mm. 20 (TN17b).

*Stratum typicum*: biomicritic limestones of the Zalumah Formation, sample TN17b.

*Type locality:* Thaytiniti, near Salalah, Sultanate of Oman.

*Age*: Priabonian (or early Rupelian).

*Additional occurrence:* Haluf near Salalah, Sultanate of Oman, same formation and age, sample HF1d (2 paratypes).

*Name*: Referring to the Arabian origin.

*Diagnosis*: as for *Arabiella* nov. gen. (only species).

*Remarks*: The two specimens from Haluf differ somewhat from those from the type locality Thaytiniti by being more slender, and having a slightly smaller aperture. The small sample size, however, is insufficient to establish a taxonomically relevant differentiation between these populations. The degree of variation is well within the range admitted in other hydrobioid species, e.g. *Lutetiella* *conica* (Prévost, 1821) and *L.* *hartkopfi* Kadolsky, 2015, which also show that the difference between individuals from the same location is greater than the difference between the four representatives of the two populations of *Arabiella arabica*.

**Genus** ***Pyrgulella*** **Harzhauser, Neubauer and Kadolsky nov. gen.**

*Type species:* *Pyrgulella parva* nov. sp.; Eocene, Priabonian (or early Rupelian), Sultanate of Oman.

*Diagnosis*: Small, conic shells with low, convex and granulose protoconch with planispiral initial part and two prominent keels on teleoconch whorls, the upper one being most prominent and coinciding with the periphery. Upper half of the whorls forming a steep sutural ramp. Aperture elongate-ovoid, adapically angulated, higher than wide; peristome concave prosocline, abapically convex. Inner lip slightly thickened; without umbilicus.

*Name*: Referring to the similarity with the extant European *Pyrgula* De Cristofori and Jan, 1832.

*Included species:* Only the type species is known so far.

*Remarks*: The systematic position of this genus is debatable and we tentatively place it within Hydrobiidae sensu lato. The monotypic genus *Sellia* de Raincourt, 1884, which was widespread during the middle and late Eocene of the eastern Atlantic (France and England; Wenz 1926), is one of the two comparable genera in the European Palaeogene. The type species *Sellia pulchra* de Raincourt, 1884 is also characterised by a prominent keel, but differs from the Omani shell in its broader outline, the wider aperture, a shallow sinus in the adapical part of the peristome, the presence of an umbilical chink and the absence of any additional spiral sculpture. (*Sellia miocaenica* Kókay, 2006, from the lower Miocene of Hungary, certainly does not belong to *Sellia* given its spiral grooves, the subcircular aperture and the ovate last whorl). The second genus is *Pseudopyrgula* Wenz, 1928, with the type species *P. sturi* (Bittner, 1884), which occurs with several species in the upper Eocene to Oligocene freshwater deposits of Trbovlje and Socka in Slovenia. Most species are strongly elongate but *P. carniolica* (Bittner, 1884) is reminiscent of the Omani shell in outline. A closer relation, however, is unlikely as all *Pseudopyrgula* species are much larger and are characterised by a single, very prominent keel, which appears close to the lower suture. The American genus *Goniobasis* Lea, 1862 (Pleuroceridae, Cerithioidea) may develop very similar morphologies, such as the Eocene *Goniobasis tenuicarinata* (Meek and Hayden, 1857), which, however, is much larger, has more whorls and a more rounded outer lip.

*Pyrgulella* is reminiscent of the late Miocene to recent European *Pyrgula* De Cristofori and Jan, 1832. A close relation, however, can be excluded based on the much higher number of whorls, the rather smooth and pointed protoconch (Szarowska 2006) and the narrower inner lip of *Pyrgula*. Sculpture and a planispiral protoconch are comparable with some genera of the Iravadiidae as revised by Ponder (1984) but the sculptured protoconch and the simple aperture differ from those of iravadiids. Moreover, the assumed freshwater environment would contradict the occurrence of this brackish-marine group. The absence of any axial sculpture on the protoconch and early teleoconch does not support a placement in a thiarid genus, such as *Melanoides* Olivier, 1804 and allies (see Bandel and Kowalke 1997; Harzhauser et al. 2015). Some Triculinae genera (Pomatiopsidae) with strong spiral sculpture, such as *Neoprososthenia* Davis and Kuo in Davis et al., 1981, *Karelainia* Davis, 1979 and *Robertsiella* Davis and Greer, 1980, are also similar to the Omani shell but differ in their wider and sub-circular ovate aperture.

***Pyrgulella parva*** **Harzhauser, Neubauer and Kadolsky nov. sp.**

Figure 5a

**Fig. 5:**
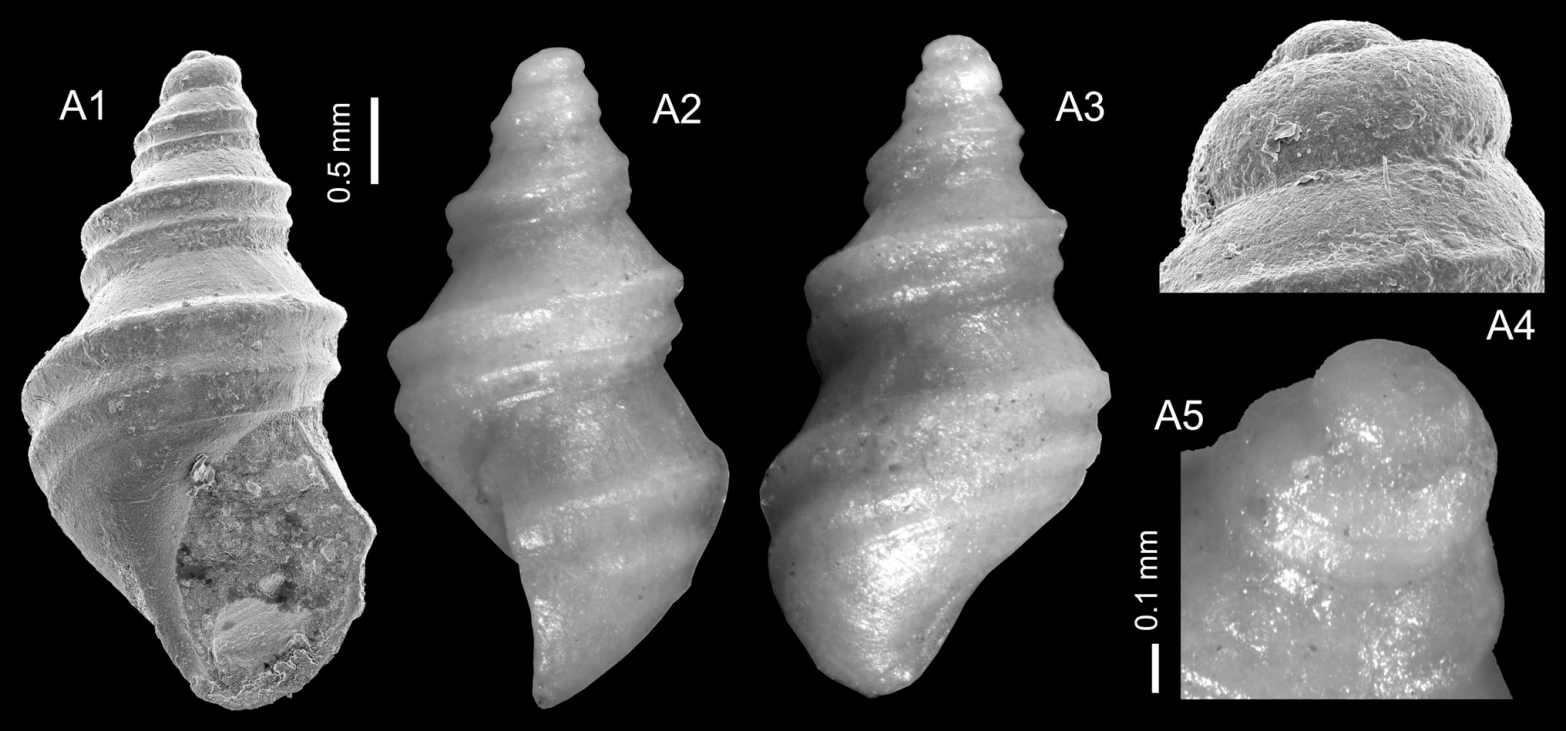
Hydrobiidae. **a** *Pyrgulella parva* Harzhauser, Kadolsky and Neubauer nov. sp. Holotype, TN3a; ONHM/TN/0017

*Holotype*: Fig. 5a, ONHM/TN/0017, height: 3.8 mm, diameter: 2.0 mm (TN3a).

*Stratum typicum*: Biomicritic limestones of the Zalumah Formation.

*Type locality*: Thaytiniti near Salalah, Sultanate of Oman.

*Age*: Priabonian (or early Rupelian).

*Name*: Referring to the small size (Latin parvus = small).

*Description*: Tiny, conic shell comprising 3.5 teleoconch whorls with two characteristic sharp keels, one at the periphery and one below; apical angle c. 40°. Protoconch consisting of about 1.7 broad and convex whorls of c. 450 μm diameter with sunken, planorbid initial part and high second whorl. Surface of protoconch not well preserved, but apparently granulose or malleate. Teleoconch whorls develop a steep sutural ramp delimited by the peripheral keel; later a weak spiral cord occurs at the lower suture. Faint spiral threads comprise the microsculpture, being best developed on the base. In addition, very delicate, weakly concave prosocline growth lines form the axial sculpture, which is best developed in the spiral concavity and the ramp. Base slowly contracting, bearing two weak spiral cords below the peripheral keel. Aperture elongate-ovate, moderately wide, adapically angulated and basally convex without siphonal incision. Columella concave with thin inner lip passing into a delicate parietal callus.

*Remarks*: See discussion on *Pyrgulella*.

*Distribution*: Only known from Thaytiniti.

Superfamily Littorinoidea Children, 1834

Family Pomatiidae Newton, 1891 (1828)

**Genus** ***Omanitopsis*** **Harzhauser and Neubauer nov. gen.**

*Type species:* *Cyclotopsis praecursor* Neubert and Van Damme, 2012. Eocene, Priabonian (or early Rupelian), Sultanate of Oman.

*Diagnosis*: Small *Cyclotopsis*-like, turbiniform shells with variable but elevated spire and evenly convex teleoconch whorls, separated by incised sutures. Sculpture consisting of spiral cords, which may be prominent or nearly obsolete. Aperture prosocline, semi-circular with slightly flaring and continuous peristome. Umbilicus deep and funnel-shaped. Sub-circular operculum with shallow-concave inner surface and prominent spiral lamella on outer surface with interspaces.

*Included species:* *Omanitopsis praecursor* (Neubert and Van Damme, 2012) and *Omanitopsis vandammei* nov. sp., both from the upper Eocene of the Sultanate of Oman.

*Name*: Referring to the Sultanate of Oman and the extant *Cyclotopsis* Blanford, 1864, which might be closely related.

*Remarks*: The generic placement of the type species was controversial. Neubert and Van Damme (2012) placed it in the extant *Cyclotopsis* Blanford, 1864 based on the overall similarity in shape and sculpture and especially in respect to the multispiral opercula. *Cyclotopsis* is confined to the Indian subcontinent (Neubert 2009) and, therefore, Pickford et al. (2014) doubted the generic identification and proposed a relation to the African genus *Tropidophora* Troschel, 1847. In our opinion, both placements are doubtful. A close relation to *Tropidophora* can be excluded based on the circular, multispiral operculum with the spiral ridge in the Omani species forming a sharp lamella. *Tropidophora*, in contrast, forms a paucispiral and smooth operculum with alate area (see Emberton et al. 2010; Wilmsmeier and Neubert 2012; Griffiths and Herbert 2013). The operculum of *Cyclotopsis* is indeed very similar to that of the Omani species. A constant difference, however, is the smooth interspace between the spiral ridge in the Omani species, whereas Neubert (2009) in his revision of *Cyclotopsis* considered sharp, straight lamellae within the interspace to be a characteristic feature for all species of that genus.

The pointed and bulbous protoconch is reminiscent of the African cyclophorid genus *Cyathopoma* Blanford and Blanford, 1861, which can be excluded based on its deeply concave, multispiral operculum with a much higher number of revolutions (see Emberton 2003; Emberton et al. 2010; Rowson et al. 2010).

The two species of *Omanitopsis* are similar to *Bembridgia cincta* (Edwards, 1852), the type species of *Bembridgia* Fischer, 1885, from the upper Eocene Bembridge Beds of England. The similarities include the shell shape, size range, and the morphologically very differentiated operculum: on the outside, the outer zone of the opercular whorls is raised and is delimited at its inner margin by a keel; in side view the margin has three keels, of which the middle one is most prominent. Nevertheless, the operculum of *Bembridgia cincta* differs in its higher number of whorls. Moreover, *B. cincta* differs from the *Omanitopsis* species by its slightly larger size (height:width is up to 11.0 mm:14.5 mm) due to the additional growth of up to 1/2 whorl, the appearance of secondary spirals on the late teleoconch whorls, the increasing strength of spirals in the umbilicus and a different tendency to reduce spiral sculpture: whereas in *O. praecursor* all spirals are affected by reduction, in *B. cincta* reduction occurs in the subsutural zone and on the umbilical side except in the umbilicus itself. *B. elegantilites* (Boubée, 1831) (syn. *Cyclostoma coquandi* Mathéron, 1843, *Cyclostoma excavatum* de Serres, 1844) is very similar and perhaps conspecific; it occurs in southern France. Both the *Bembridgia* species are associated with the mammal biozone MP19 (Priabonian). Thus, they are of similar age to the *Omanitops* species and very likely closely related.

Only two additional pomatiid genera are known from the Tethyan Eocene: *Procyclotopsis* Wenz, 1924, with the Italian type species *Cyclotus laevigata* Sandberger, 1870, and *Palaeocyclotus* Fischer, 1885, with the Italian type species *Cyclotus exaratus* Sandberger, 1870. *Palaeocyclotus* is low turbiniform and develops a highly multispiral operculum, excluding any relation to *Omanitopsis*. *Procyclotopsis*, as understood by Wenz (Wenz 1923a, b; as *Cyclotellina*; see Harzhauser et al. 2014b), comprises Bartonian species from Italy. The type species *C. laevigata* is high turbiniform with deep sutures, smooth teleoconch (except for a striate first teleoconch whorl) and has a strongly pointed protoconch; the terminal part of the last whorl is detached from the base or at least attached far below the periphery from the preceding whorl. Its multispiral operculum has distinct radial lamellae. A close relation with *Omanitopsis* can thus be excluded.

***Omanitopsis praecursor*** **(Neubert and Van Damme,** 2012)

Figures 6a–c, 7e

**Fig. 6:**
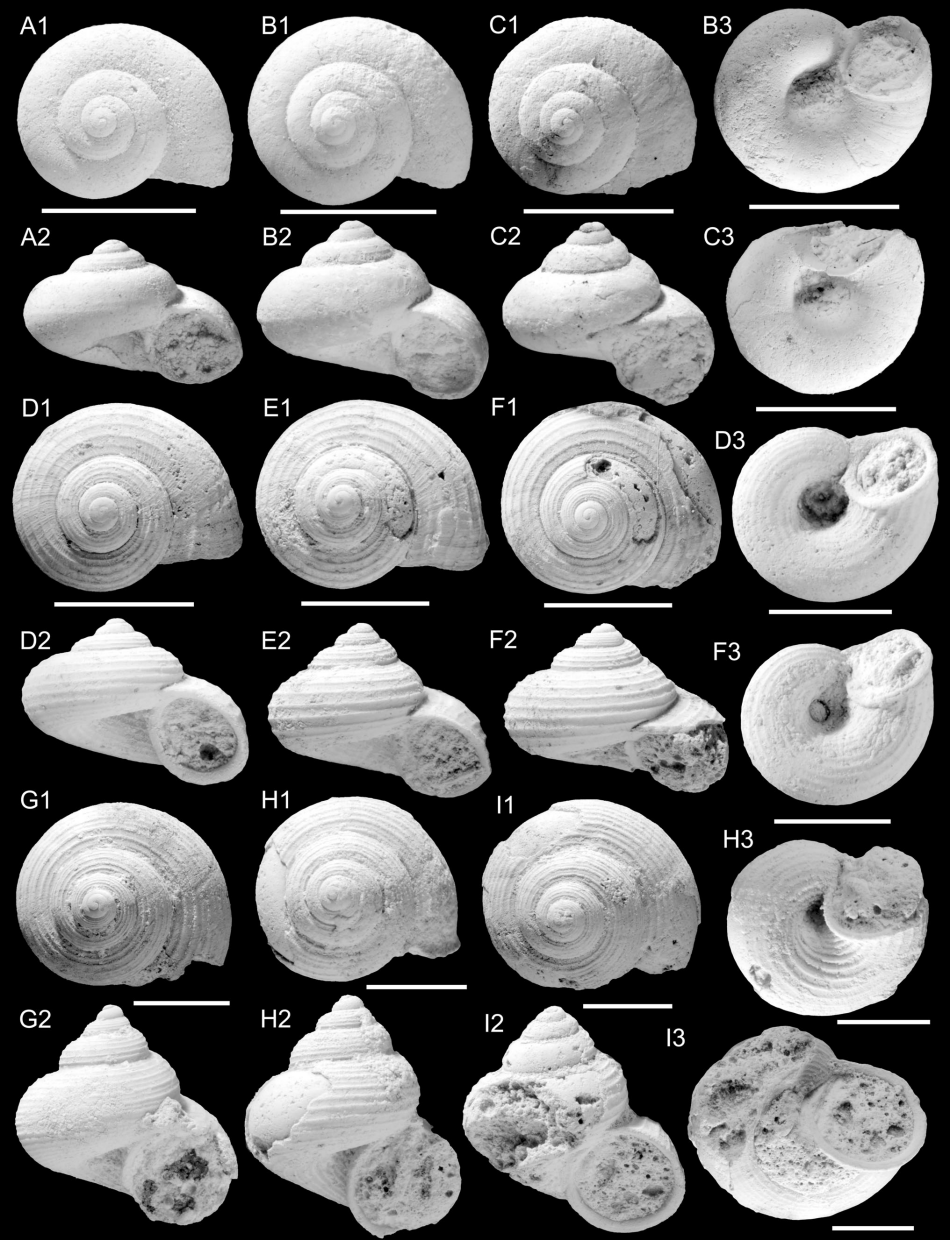
Pomatiidae. **a**–**c** *Omanitopsis praecursor* (Neubert and Van Damme, 2012) **a** TN3a; ONHM/TN/0018. **b** TN3a; ONHM/TN/0019. **c** TN3a; ONHM/TN/0020. **d**–**f** *Omanitopsis vandammei* Harzhauser and Neubauer nov. sp. **d** Holotype, HF4b; ONHM/HF/0003. **e** Paratype, HF4b; ONHM/HF/0004. **f** Paratype, HF4c; ONHM/HF/0005. **g**–**i** *Procyclotopsis eocenica* Harzhauser and Neubauer nov. sp. **g** Holotype, HF4b; ONHM/HF/0006. **h** Paratype, HF4b; ONHM/HF/0007. **i** Paratype, HF4b; ONHM/HF/0008. *Scale bar* 5 mm

**Fig. 7:**
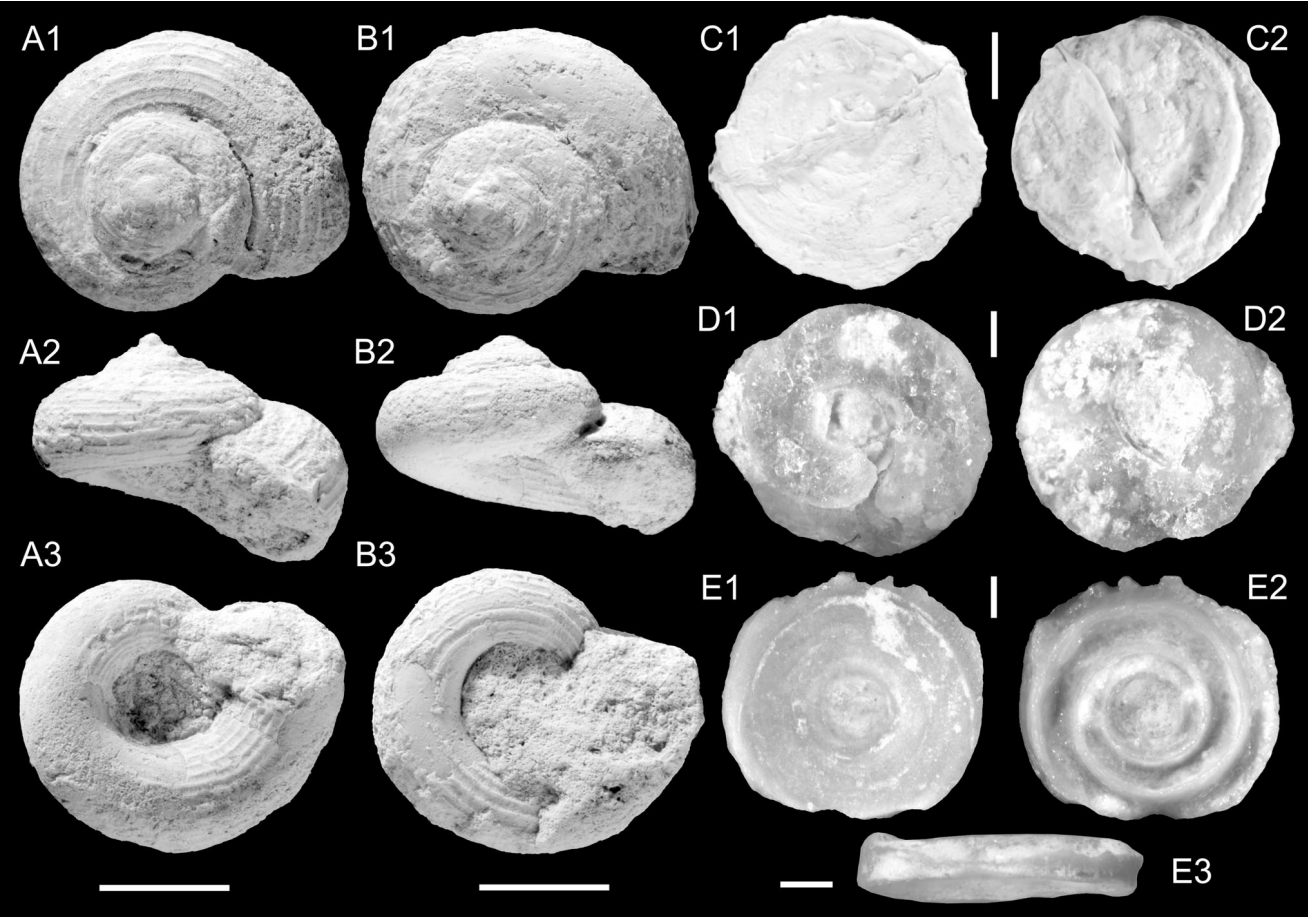
Pomatiidae. **a**–**c** *Palaeocyclotus kuehschelmi* Harzhauser and Neubauer nov. sp. **a** Holotype, TQ3; ONHM/TQ/0001. **b** Paratype, TQ3; ONHM/TQ/0001. **c** operculum probably of *Palaeocyclotus kuehschelmi* TQ3; ONHM/TN/0003. **d** operculum probably of *Procyclotopsis eocenica* Harzhauser and Neubauer nov. sp. HF4b; ONHM/HF/0009. **e** *Omanitopsis praecursor* (Neubert and Van Damme, 2012), operculum, TN3a; ONHM/TN/0021. **a**–**b** *Scale bar* 5 mm. **c**–**e** *Scale bar* 1 mm

* 2012 *Cyclotopsis praecursor* Neubert and Van Damme: 10, Fig. 8 (non Fig. 9).

**Fig. 8:**
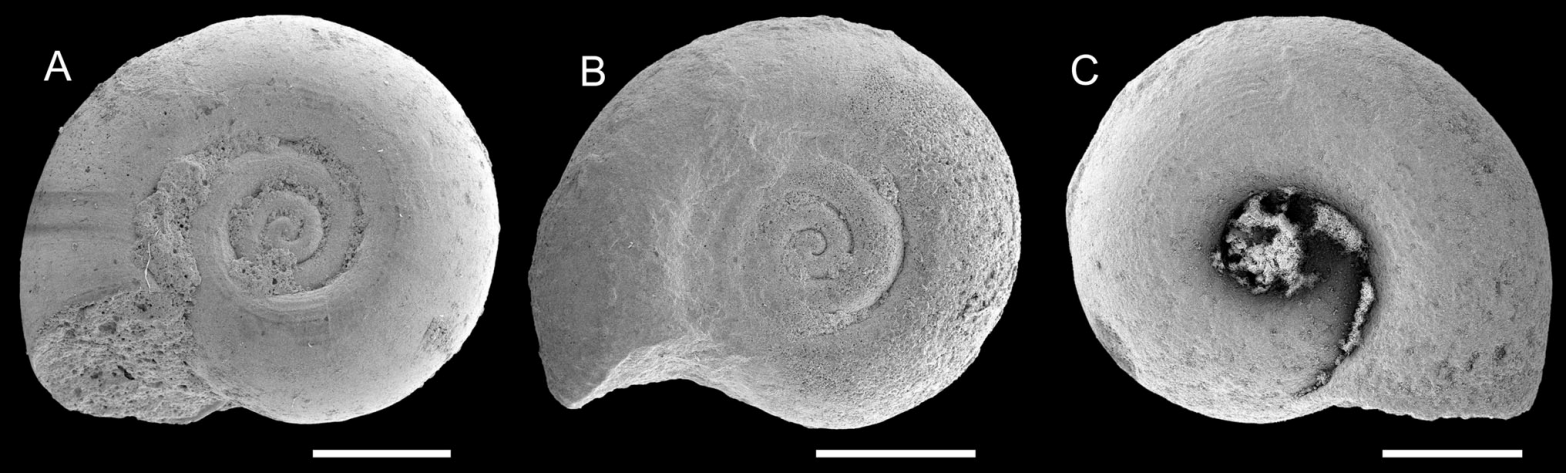
Planorbidae. **a**–**c** *Planorbarius* sp. **a** TN17b; ONHM/TN/0022. **b** TN3c; ONHM/TN/0023. **c** TN9; ONHM/TN/0024. *Scale bar* 1 mm

**Fig. 9:**
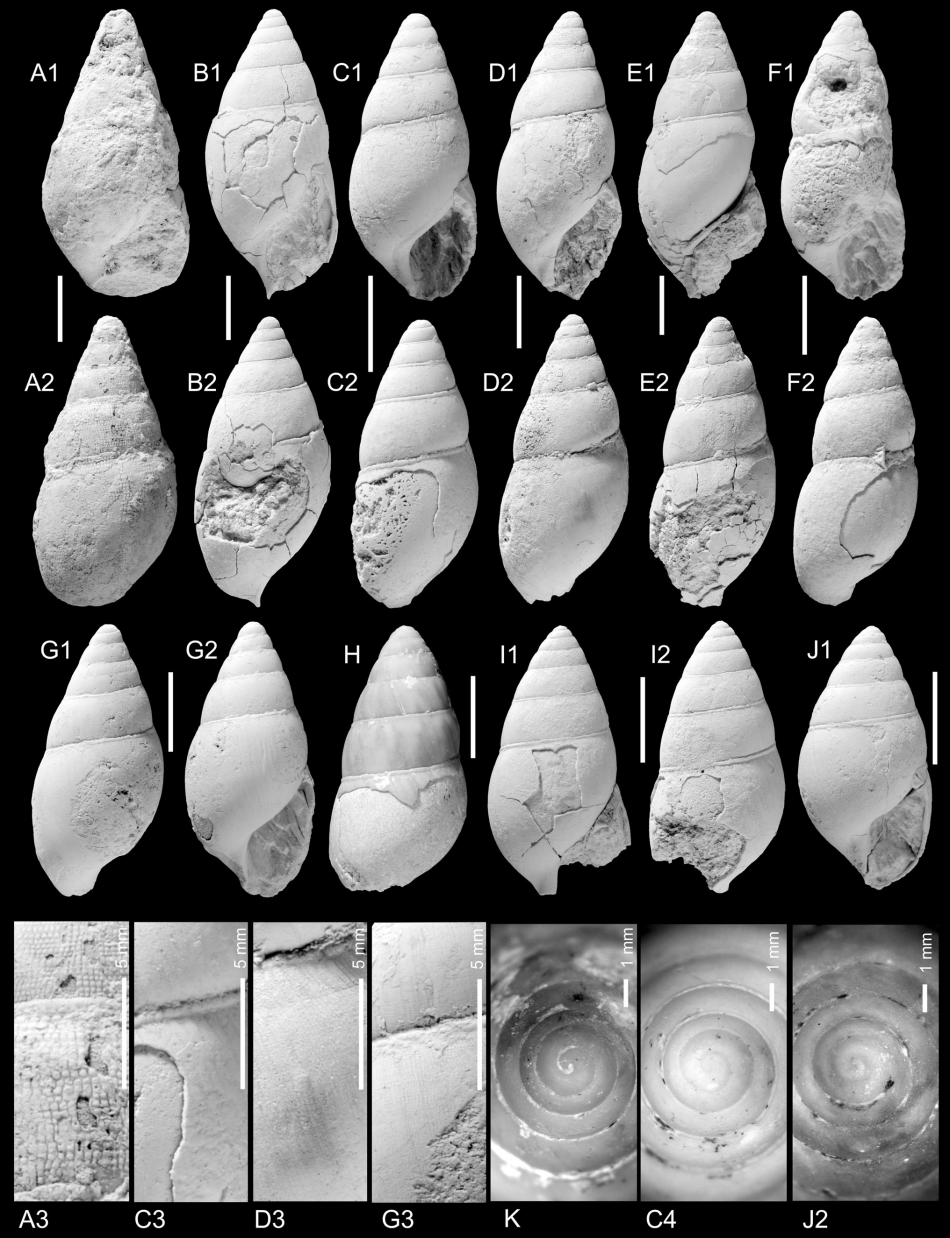
Vidaliellidae. **a** *Arabicolaria sculpturata* (Neubert and Van Damme, 2012), TQ2; ONHM/TQ/0004. **b** *Arabicolaria arabica* Harzhauser and Neubauer nov. sp. Holotype, TN3a; ONHM/TN/0025. **c**–**f** *Arabicolaria omanensis* (Neubert and Van Damme, 2012). **c** TN3a; ONHM/TN/0026. **d** TN3a; ONHM/TN/0027. **e** TN15c; ONHM/TN/0028. **f** TN15c; ONHM/TN/0029. **g** *Pacaudiella omanica* Harzhauser and Neubauer nov. sp., Holotype, TN15c; ONHM/TN/0030. **h**–**i** *Pacaudiella flammulata* Harzhauser and Neubauer nov. sp. **h** Holotype, TN3a; ONHM/TN/0032. **i** Paratype, TN3a; ONHM/TN/0033. **j** *Pacaudiella* cf. *flammulata* Harzhauser and Neubauer nov. sp., TN3a; ONHM/TN/0031. **k** *Arabicolaria arabica* Harzhauser and Neubauer nov. sp. Paratype, TN15c; ONHM/TN/0034. *Scale bar* 5 mm if not stated otherwise

2014 *Tropidophora praecursor*.—Pickford et al.: 95, 96 (pars).

*Material*: 412 (TN3a, illustrated specimens: ONHM/TN/00018–ONHM/TN/0021), 164 (TN8), 129 (TN9), 429 (TN12), 5 (TN15b), 355 (TN17b); 12 opercula (TN3a).

*Measurements*: mean height: 6.5 mm (*σ* = 0.61), mean diameter: 8.6 mm (*σ* = 0.62) (*n* = 12).

*Description*: Small, turbiniform shells of four evenly convex teleoconch whorls separated by deep, but narrow sutures; spire height slightly variable. Large protoconch comprising about one protruding and bulbous whorl of ca. 1.1 mm diameter; transition into teleoconch indistinct. Surface is smooth in most specimens from localities TN7 and TN9; only a few specimens display five very weak spiral cords on the second teleoconch whorl, fading out within the third whorl. In sample TN8 the spiral sculpture is more prominent and is recognisable even on the last whorl. Aperture nearly circular with weak adapical angulation; peristome slightly flaring, continuous with inner lip. Apertural margin attached close below periphery, which may form a very indistinct angulation. Umbilicus deep and funnel-shaped with smooth walls. Twelve opercula are available ranging between 3.0 to 3.3 mm in diameter and ca. 0.5 mm in thickness. They are sub-circular with central nucleus; inner surface shallow-concave and smooth with weak opercular suture; alate area very narrow. Outer surface flat with a prominent spiral ridge of 3.5 whorls formed by a solid lamella; interspaces without discernible growth lamellae. Inner and outer surface not elevated in lateral view; opercular edge with three keels.

*Remarks*: Neubert and Van Damme (2012) based this species on a rather smooth, turbiniform specimen with attached operculum. In addition, they considered a strongly sculptured and more trochiform specimen (Neubert and Van Damme 2012, Fig. 9) to be conspecific with *O. praecursor*. They interpreted the smoother shells to represent mainly internal casts lacking any sculpture. The rich material available to us from Thaytiniti (>400 specimens) contains numerous well-preserved specimens with perfect calcitic pseudomorphoses of the shells. Although there is some variability in sculpture (as discussed above), none of the specimens agrees with the sculpture type shown in Fig. 9 of Neubert and Van Damme (2012). Therefore, we propose that this shell belongs to a separate species discussed below.

*Distribution*: Only known from Thaytiniti.

***Omanitopsis vandammei*** **Harzhauser and Neubauer nov. gen. nov. sp.**

Figures 6d–f

2012 *Cyclotopsis praecursor* Neubert and Van Damme: 10, Fig. 9.

2014 *Tropidophora*.—Pickford et al.: 95, Fig. 3c.

*Holotype*: Fig. 6d, ONHM/HF/0003, height: 6.8 mm, diameter: 8.9 mm (HF4b).

*Paratype*: Fig. 6e, ONHM/HF/0004, height: 7.0 mm, diameter: 9.0 mm (HF4b).

*Paratype*: Fig. 6f, ONHM/HF/0005, height: 7.0 mm, diameter: 9.0 mm (HF4c).

*Additional material:* 64 (HF1a), 28 (HF3b), 129 (HF4b), 99 (HF4c), 64 (TN17b).

*Size range*: mean height: 6.9 mm (*σ* = 0.47), mean diameter: 9.6 mm (*σ* = 0.61) (*n* = 12).

*Stratum typicum*: Biomicritic limestones of the Zalumah Formation.

*Type locality*: Haluf near Salalah, Sultanate of Oman.

*Age*: Priabonian (or early Rupelian).

*Name*: In honour of Dirk Van Damme (University Gent, Belgium) and his contributions to African palaeo-malacology.

*Description*: Bulbous protoconch 1.4 mm in diameter, comprising 1.5 convex whorls with weakly malleate surface; transition into teleoconch marked by onset of five to six spiral cords. The spiral cords are prominent, rather narrow, rounded and evenly spaced; the wide interspaces bear densely spaced axial threads. The 3–3.5 teleoconch whorls are usually evenly convex apart from some specimens with slight angulation. The spiral cords also appear on the umbilical side of the shell, but become weaker towards the wide, perspectivic umbilicus. Aperture margin attached below periphery, prosocline, subcircular, abruptly flaring with continuous and thickened peristome. Operculum unknown.

*Remarks*: Neubert and Van Damme (2012) intermingled this species with their new species “*Cyclotopsis*” *praecursor*. Despite the similarities in outline, we consider it to be a distinct species, which differs from *Omanitopsis praecursor* in the much stronger sculpture, the presence of axial threads and the much thicker and well-defined peristome. Even poorly preserved specimens of *Omanitopsis vandammei* can be easily recognised and do not show a tendency to reduce the sculpture on the last whorl. *Omanitopsis vandammei* is mainly found at Haluf and *O. praecursor* occurs at Thaytiniti. The two species co-occur in only a single sample from Thaytiniti (TN17b). Most probably, this pattern indicates a minor stratigraphic and/or ecological difference between the localities.

*Distribution*: Known from the Haluf area and from Thaytiniti.

**Genus** ***Procyclotopsis*** **Wenz,** 1924

*Type species:* *Cyclotus laevigatus* Sandberger, 1870; by typification of replaced name *Cyclotellina* Wenz 1923 (original designation), non Cossmann 1886. *Cyclotellinodes* Strand 1928 is an unnecessary replacement name; *Procyclotella* Wenz 1938–1944 is a lapsus; see also Harzhauser et al. 2014b.

***Procyclotopsis eocenica*** **Harzhauser and Neubauer nov. sp.**

Figures 6g–i, 7d

2014 *Tropidophora* sp. 2.—Pickford et al.: 96.

*Holotype*: Fig. 6g, ONHM/HF/0006, height: 12.5 mm, diameter: 12.0 mm (HF4b).

*Paratype*: Fig. 6h, ONHM/HF/0007, height: 12 mm, diameter: 11.5 mm (HF4b).

*Paratype*: Fig. 6i, ONHM/HF/0008, height: 12.5 mm, diameter: 13.5 mm (HF4b).

*Additional material*: 43 (HF1d), 6 (HF4b), 9 (HF4c).

*Size range*: mean height: 13.0 mm (*σ* = 0.89), mean diameter: 12.7 mm (*σ* = 1.1) (*n* = 8).

*Stratum typicum*: Biomicritic limestones of the Zalumah Formation.

*Type locality*: Haluf near Salalah, Sultanate of Oman.

*Age*: Priabonian (or early Rupelian).

*Name*: Referring to the Eocene age of the Zalumah Formation.

*Description*: Medium-sized turbiniform shell, only slightly wider than high. Protoconch comprising about 1.7 whorls of ca. 1.8 mm diameter, with very low initial part, followed by a high and convex whorl; surface probably slightly granulose. Transition into teleoconch marked by onset of six broad spiral cords with convex tops and slightly broader and smooth interspaces. Teleoconch consisting of 3.5 evenly convex, shoulderless whorls with deeply incised sutures. Number of spiral cords increases to seven on penultimate whorl and to ca. 25 on the last whorl including the base. The spiral cords become weaker along the periphery and are prominent again in the circumumbilical area. Umbilicus moderately wide, deeply conical, and narrowed by the columellar lip; umbilical wall covered by spiral cords. Aperture subcircular and weakly prosocline with abruptly flaring peristome; columellar lip and basal lip reflected, broad and even widening at the attachment to the base. Spiral sculpture of the teleoconch stops abruptly before the peristome.

*Remarks*: This species clearly differs from the two co-occurring *Omanitopsis* species by its much higher protoconch, the much larger size and the higher spire. The generic placement is questionable; the Omani species is highly reminiscent of *P. obtusicosta* (Sandberger, 1870) from the Eocene of the Italian Veneto, which differs mainly in its narrower peristome and higher number of spiral cords. Wenz (1923a, b) listed the Italian species within his new genus *Cyclotellina* (non *Cyclotellina* Cossmann, 1886), but designated *Cyclotus laevigata* Sandberger, 1870 as the type species. *Procyclotopsis laevigata* is characterised by its pointed protoconch and a teleoconch which is nearly smooth apart from spiral threads on the first spire whorl. Therefore, it is questionable whether the strongly sculptured Italian species, listed by Wenz (1923a, b, 1942), are congeneric with *P. laevigata*. As a revision of this group is beyond the scope of this paper, we provisionally place the Omani shell in *Procyclotopsis*. *Procyclotopsis* *galianae* (Esu, 1984) from the upper Eocene or lower Oligocene of Majorca, has a higher last whorl and a broader spire.

A singly, very poorly preserved operculum is available from sample HF1d (Fig. 7D; ONHM/HF/0009). In respect to size, it might belong to this species and represents a robust, rather thick, paucispiral operculum with narrow alate area lacking a spiral ridge.

*Distribution*: Only known from the Haluf area.

**Genus** ***Palaeocyclotus*** **Fischer,** 1885

*Type species*: *Cyclotus exaratus* Sandberger, 1870; by monotypy. Eocene, Italy.

***Palaeocyclotus kuehschelmi*** **Harzhauser and Neubauer nov. sp.**

Figure 7a–c

*Holotype*: Fig. 7a, ONHM/TQ/0001, height: 9 mm, diameter: 13.5 mm (TQ3).

*Paratype*: Fig. 7b, ONHM/TQ/0002, height: 7 mm, diameter: 13.5 mm (TQ3).

*Additional material:* 142 (TQ2), 27 (TQ3).

*Size range:* mean height: 7.7 mm (*σ* = 0.87), mean diameter: 13.2 mm (*σ* = 0.51) (*n* = 12).

*Stratum typicum:* Biomicritic limestones of the Zalumah Formation.

*Type locality:* Taqah near Salalah, Sultanate of Oman.

*Age*: Priabonian (or early Rupelian).

*Name*: In honour of Horst Kühschelm, volunteer at the Geological-Palaeontological Department of the Natural History Museum Vienna.

*Description*: Low turbiniform shell with very low spire and large and wide last whorl. Protoconch poorly preserved, consisting of approximately one convex and pointed whorl. Teleoconch comprising 3.5 evenly convex whorls with deep sutures. Sculpture consisting of prominent spiral cords with an indistinct secondary spiral thread intercalated; about 20 spiral cords occur on the last whorl. Delicate growth lines are visible between the spiral cords. Umbilicus very wide with prominent spiral cords on the umbilical wall. Aperture circular, prosocline with only weakly flaring peristome. The terminal part of the last whorl grows in the abapical direction relative to the suture. Consequently, the apertural margin is even detached from the base in some specimens. A single operculum is available, which might belong to this species (ONHM/HF/0003). It is robust, thick, multispiral with at least six revolutions; inner side shallow-concave and smooth; outer surface poorly preserved, but with prominent spiral ridge subparallel to margin.

*Remarks*: The Omani shells are morphologically very close to *Palaeocyclotus exaratus* (Sandberger, 1870) from the middle Eocene of Italy, Dalmatia, and Switzerland (Oppenheim 1890; Locard 1893; Wenz 1923b), which thus far is the only species placed in *Palaeocyclotus*. The two species are identical in size and outline and also agree in general sculpture; both develop a multispiral operculum with about six revolutions. Separation from *P. exaratus* is based on the wider umbilicus and the lower number of spiral cords in the Omani species. A characteristic feature of this genus, apart from the low turbiniform shell, is the abapically growing terminal part of the last whorl with a detached aperture and the prominent spiral sculpture of the umbilical walls.

*Distribution*: Only known from Taqah.

Clade Panpulmonata Jörger et al., 2010

Order Hygrophila Férussac 1822

Superfamily Planorboidea Rafinesque, 1815

Family Planorbidae Rafinesque, 1815

**Genus** ***Planorbarius*** **Duméril,** 1806

*Type species:* *Helix cornea* Linnaeus, 1758; by subsequent monotypy by Froriep (1806). Recent, Europe.

***Planorbarius*** **sp.**

Figure 8a–c

*Material*: 3 (TN3A; ONHM/TN/0022–ONHM/TN/0024), 3 (TN3c), 1 (TN9), 3 (TN17b).

*Description*: Regularly coiled, planispiral shell with up to 3.6 high and convex whorls. Convexity has its maximum around whorl centre; whorls are slightly flattened on both apical and umbilical sides. Apical depression broad and shallow; umbilical depression more narrow and deep. Whorls overlap ca. 15 % of preceding ones; they are separated by marked sutures. Shells are unevenly covered with faint to distinct spiral striae.

*Remarks*: The planorbids of Thaytiniti are represented by a single, rare species only. Its full size cannot be estimated, since apparently only juvenile specimens are preserved. The maximum diameter among the available specimens is 4.0 mm, the maximum height is 1.8 mm. The typical shape and striation argue for a classification as *Planorbarius* (see, e.g., Harzhauser et al. 2014a, b). Identification at the species level is, however, not possible. We likely deal with juveniles only and even adult specimens of *Planorbarius* species are often difficult to differentiate. A similar species is *Planorbarius crassus* (Serres, 1844) from the upper Eocene to lower Oligocene of France. It shares the high, convex profile, the wide, shallow apical depression, and the typical striation (Serres 1844: p. 178, pl. 12, Fig. 5; Maillard 1892). *Planorbarius choffati* (Maillard, 1886) from the upper Eocene to lower Oligocene of Switzerland can be distinguished by the deep, narrow apical and umbilical depressions and the very distinct growth lines (Maillard 1886: p. 11, pl. 1, Figs. 4–5). *Planorbarius euomphalus* (Sowerby, 1818) from the Eocene to lower Oligocene of Great Britain and France has a flattened umbilical side, a wide, but deep apical depression and an asymmetric profile with a weak keel (cf. Sandberger 1873; classification following Le Renard and Pacaud 1995). *Planorbarius junici* Marquet, Lenaerts, Karnekamp and Smith, 2008, from the Rupelian of Belgium, is another comparable species, which differs in its weaker striation and the wider whorls.

Superorder Eupulmonata Haszprunar and Huber, 1990

Order Stylommatophora Schmidt, 1855

incertae sedis

Family Vidaliellidae Nordsieck, 1986

**Genus** ***Arabicolaria*** **Harzhauser and Neubauer nov. gen.**

*Type species:* *Limicolaria omanensis* Neubert and Van Damme, 2012. Eocene, Priabonian (or early Rupelian), Sultanate of Oman.

*Diagnosis*: Elongate bulimoid shells with dome-shaped protoconch, weakly convex spire whorls, and more or less convex and ovoid last whorl. A narrow and prominent subsutural spiral cord is typical. Aperture elongate-ovoid with shallow canal and adapical angulation; a weak parietal callus may be present; columella truncated; anomphalous. Sculpture consisting of densely spaced axial riblets crossed by spiral grooves, resulting in a faint to very coarse granulation (mostly in the upper half of the whorls).

*Included species:* *Arabicolaria omanensis* (Neubert and Van Damme, 2012) and *A. arabica* nov. sp., both from the upper Eocene of the Sultanate of Oman. Co-occurring *Achatina sculpturata* Neubert and Van Damme, 2012 is tentatively also transferred into this genus.

*Name*: A mixture of Arabia and *Limicolaria*, referring to the geographical distribution and the *Limicolaria*-like outline.

*Remarks*: Neubert and Van Damme (2012) and Pickford et al. (2014) treated two species from the Omani Eocene with overall achatinid morphology as Achatinidae. This exclusively African group is tightly linked to Subulinidae and seems to have split from subulinids quite recently with low genetic divergence (see Wade et al. 2006). Therefore, the presence of the two modern Achatinidae genera *Achatina* and *Limicolaria* already 35 million years ago is not very likely. Moreover, Mead (1992) suggested that the modern Achatinidae originated from a West African centre of origin, based on the more advanced genital structures in South and Eastern African groups. This scenario of a geologically rather young (Tillier 1989) dispersal from west to east, which seem to be supported by molecular data (Fontanilla 2010), would clearly exclude a close relation of the Omani Eocene fossils with any modern genera. In contrast, Fred Naggs (pers. comm. 2014), who was co-author of Wade et al. (2006), indicated that the existence of the family Achatinidae during the Eocene cannot be excluded based on their molecular phylogeny. Doubts about the achatinid relations also derive from morphological data. All species described herein are characterised by a prominent and well-defined subsutural cord, which is atypical for Achatinidae in which the subsutural area is less defined.

Another achatinid-like group, which was well established during the Eocene in the Tethyan Realm are the Vidaliellidae Nordsieck, 1986. This group split from the Anadromidae Wenz, 1940 probably already in Cretaceous times and is treated as a full family herein. It is common in the Palaeogene of North Africa and southwestern Europe and is represented by genera characterised by a bulimoid shape and often by an achatinid-like sculpture. The genera of the group were discussed by Jodot (1957a), Wenz and Zilch (1960), Plaziat (1973) and Nordsieck (1986). The Thanetian to early Oligocene *Vidaliella* Wenz, 1940, with the Spanish type species *V. gerundensis* (Vidal, 1883), develops a low and wide aperture, nearly flat spire whorls, a canaliculate suture and a strongly thickened peristome (Plaziat 1973; Piñero 2010; Adaci 2012; Ortí and Valls 2013). These features allow a clear separation from all Omani species discussed herein. Moreover, *Vidaliella* lacks a subsutural cord [apart from an unnamed species from the Oligocene of Majorca described by Esu (1984)]. *Romanella* Jodot, 1953 (in Jodot 1953a), with the Eocene type species *Romanella hopii* (Serres, 1827) from France, has an elongate fusiform outline with high spire, but is otherwise very similar or even congeneric with *Vidaliella* especially in the flaring peristome with evenly rounded basal part (see Roman 1899; Plaziat 1973). *Vidalella darderi* (Vidal, 1917), as type species of *Vidalella* Jodot, 1957 (in Jodot 1957a), has a rather globular outline, a short spire, a thickened outer lip and a wide aperture with a *Melanopsis*-like parietal pad.

In contrast, *Vicentinia* Jodot, 1957 (in Jodot 1957a), with the Italian Eocene type species *Bulimulus eocaenus* Oppenheim, 1890, is morphologically much closer to the Omani species. *Vicentinia* had a Western Tethyan distribution and develops medium-sized bulimoid shells with slightly reflected inner lip with a chink-like umbilicus. In their overall shape, some Omani shells are reminiscent of *V. timhaditensis* Jodot, 1957 (in Jodot 1957b) and *V. eocenica* but differ in their higher base and the consequently narrower aperture. All other species described by Jodot (1957b) are either much more elongate (*V. gracilis*, *V. acuta*) or very stout (*V. salvini*). A general difference, however, is the absence of a subsutural cord and the rapidly contracting base of all *Vicentinia* species, which seems to exclude a close relation. “*Clavator* (*Leucotaenius*)” sensu Jodot (1938, 1953b) (non Martens in Albers 1860) from the northwestern African Palaeogene represents a further vidaliellid genus, probably closely related to *Romanella* or *Vicentinia*, with stout bulimoid shape and somewhat detached, strongly angulated apertural margin. Like *Vicentinia* it is similar to some of the stout species from Oman, but lacks a distinct subsutural cord. Finally, *Procerastus* Wenz, 1924, might belong to the Vidaliellinae as well. Its Eocene type species *Procerastus vicentinus* (Oppenheim, 1890) from Italy is characterised by a continuous parietal callus connecting the inner and outer lips. This feature seems to be absent in *Vicentinia* and in the Omani species.

Thus, based on the overall similarities with other vidaliellid genera and in respect to the widespread and frequent occurrence of the Vidaliellidae in the Tethyan Palaeogene we consider that the bulimoid Omani species are members of this family.

***Arabicolaria omanensis*** **(Neubert and Van Damme,** 2012 **)**

Figure 9c–f

*2012 *Limicolaria omanensis* Neubert and Van Damme: 14, Figs. 13–14.

2014 *Limicolaria omanensis*.—Pickford et al.: 95, 96.

*Material*: 4 (TN3a), 1 (TN15c).

*Measurements*: Height: 31.5 mm, diameter: 14 mm (TN3a, Fig. 9c, ONHM/TN/0026); height: 43 mm, diameter: 19 mm (TN3a, Fig. 9d, ONHM/TN/0027); largest specimen: height: 50 mm, diameter: 22 mm (TN15c, Fig. 9e, ONHM/TN/0028).

*Description*: Elongate fusiform shell of six teleoconch whorls and bulimoid spire with apical angle of ca. 45°. Dome-shaped protoconch consisting of ca. 0.75 low initial whorls forming a plane with the high and moderately convex second whorl of ca. 3 mm diameter; transition into teleoconch indistinct. Spire whorls weakly convex, with weak sutures and narrow, but prominent and well-defined subsutural spiral cord, which already appears on early spire whorls (Fig. 6c4). Sculpture consisting of densely spaced, very fine and slightly irregular axial riblets. This sculpture continuous on the last whorl, where broad and low axial ribs are formed close to the aperture and especially on the upper half of the whorl (Fig. 6c3, d3). Last whorl elongate-ovoid with slowly contracting base. Aperture elongate-ovoid; columella weakly concave, slightly twisted and truncated. Slightly thickened inner lip passing into a weak parietal callus, both being well demarcated from the base. Outer lip moderately convex and probably simple. No umbilicus or umbilical chink developed.

*Remarks*: The type material available to Neubert and Van Damme (2012) lacked large parts of the aperture. Therefore, the authors based their generic placement mainly on the teleoconch sculpture and emphasised the uncertainties. The new material from Thaytiniti suggests a truncated columella and is clearly anomphalous. Hence, a placement in the extant achatinid genus *Limicolaria* Schumacher, 1817 as defined by Pilsbry (1904) and Crowley and Pain (1970) can be excluded. This species clearly differs from co-occurring *Arabicolaria sculpturata* (Neubert and Van Damme, 2012) in the much finer sculpture, broader spire, and more elongate last whorl.

*Distribution*: Only known from Thaytiniti.

***Arabicolaria arabica*** **Harzhauser and Neubauer nov. gen. nov. sp.**

Figure 9b, k

*Holotype*: Fig. 9b, ONHM/TN/0025, height: 45 mm, diameter: 20 mm (TN3a).

*Paratype*: Fig. 9k, ONHM/TN/0034, height: 31 mm, diameter: 18 mm (TN15c).

*Additional material*: 4 (TN3a); 1 (TN15c).

*Stratum typicum*: Biomicritic limestones of the Zalumah Formation.

*Type locality*: Thaytiniti, near Salalah, Sultanate of Oman.

*Age*: Priabonian (or early Rupelian).

*Name*: Referring to the Arabian origin of the fossil.

*Description*: Stout fusiform shell comprising 6–7 whorls; protoconch starting with an immersed initial part followed by a dome-shaped and probably smooth second whorl of ca. 2.9 mm diameter; transition into teleoconch unclear. Spire conical with weakly convex whorls forming an apical angle of ca. 45°–50°. Suture narrow and incised; a slightly granular subsutural cord appears during the 3rd to 4th teleoconch whorl. Sculpture consisting of densely spaced, weakly sigmoidal-prosocline axial riblets crossed by much weaker and densely spaced spiral grooves, resulting in a finely granular pattern, which is best developed between sutural cord and periphery. Last whorl ovoid with moderately wide aperture with acute posterior angle; columella deeply concave and truncated. Inner lip forms a thin sheet expanding on the base and fades out in apical direction; no umbilicus developed.

*Remarks*: This species differs from *Arabicolaria omanensis* in its stout shell, broader spire and higher and more convex last whorl with the periphery close to the suture, whereas the periphery is much lower in *A. omanensis*. A further difference is the subsutural cord, which appears very early in *A. omanensis*, whereas the early spire whorls of *A. arabica* lack a subsutural cord.

*Distribution*: Only known from Thaytiniti.

***Arabicolaria sculpturata*** **(Neubert and Van Damme,** 2012 **)**

Figure 9a

* 2012 *Achatina sculpturata* Neubert and Van Damme: 15, Fig. 15.

2014 *Tholachatina sculpturata*.—Pickford et al.: 95.

2014 *Limicolaria*.—Pickford et al.: 95, Fig. 3b.

2014 *Achatina sculpturata*.—Pickford et al.: 96.

2014 *Limicolaria*.—Pickford et al.: 97, Figs. 5c1–c2.

*Material*: 4 (TQ2, ONHM/TQ/0004).

*Measurements*: Largest specimen; height: 43 mm, diameter: 22 mm.

*Remarks*: Only a few specimens are available of this species, and all are smaller than the 67-mm-high holotype described by Neubert and Van Damme (2012). A characteristic feature is the high, pointed, and conical spire with an apical angle of ca. 35°, the peculiar sculpture of densely spaced, axially arranged granules, and the broad subsutural cord.

Pickford et al. (2014) proposed a placement of this species in *Tholachatina* Bequaert, 1950, probably due to the modern East and South African biogeography of this group. This taxon was originally erected as subgenus of *Archachatina* Albers, 1850. The entire group has large protoconchs correlated with the production of very large eggs (Bequaert 1950). Our material lacks the protoconch and, therefore, we cannot use this feature. *Tholachatina*, however, is a subjective junior synonym of *Cochlitoma* Férussac, 1821 as their type species turned out to be congeneric (Mead 2004).

*Distribution*: Known from Taqah (this paper) and the Haluf area (Neubert and Van Damme 2012).

**Genus** ***Pacaudiella*** **Harzhauser and Neubauer nov. gen.**

*Type species*: *Pacaudiella omanica* nov. sp.; Eocene, Priabonian (or early Rupelian), Sultanate of Oman.

*Diagnosis*: Stout ovoid, bulimoid shells with moderately convex spire whorls and an ovoid last whorl; suture adjoined by a prominent subsutural spiral cord. Aperture rather narrow, ovoid with adapical angulation, shallow siphonal canal and straight, truncated columella. Inner lip reflected forming a faint umbilical chink. Sculpture ranging from nearly smooth to distinct, always consisting of faintly granulose growth lines.

*Included species*: *Pacaudiella omanica* nov. sp., *Pacaudiella eocenica* nov. sp. and *Pacaudiella flammulata* nov. sp., all from the Eocene of the sultanate of Oman.

*Name*: In honour of Jean-Michel Pacaud of the Muséum National d’Histoire Naturelle, Paris, a specialist of Eocene mollusc faunas.

*Remarks*: Its overall shape and the straight columella are reminiscent of *Vicentinia* Jodot, 1957 (in Jodot 1957a), but the presence of a prominent subsutural cord allows a clear separation from that western Tethyan genus. *Pacaudiella* differs from *Arabicolaria* in the smaller shells, the stout outline and especially in the presence of a narrow umbilical chink. Nevertheless, we consider *Pacaudiella* to belong to the same Arabian Vidaliellidae-radiation as *Arabicolaria*, based on the homologous development of the sculpture and the characteristic subsutural spiral cord.

***Pacaudiella omanica*** **Harzhauser and Neubauer nov. gen. nov. sp.**

Figure 9g

*Holotype*: Fig. 9g, ONHM/TN/0030, height: 35 mm, diameter: 16 mm (TN15c).

*Stratum typicum*: Biomicritic limestones of the Zalumah Formation.

*Type locality*: Thaytiniti near Salalah, Sultanate of Oman.

*Age*: Priabonian (or early Rupelian).

*Name*: Referring to the Sultanate of Oman.

*Description*: Medium-sized, bulimoid shell with somewhat barrel-like last whorl. Spire consisting of five moderately convex spire whorls. Protoconch unknown. Impressed suture adjoined by a narrow subsutural spiral cord. Sculpture consisting of fine, slightly irregular, and weakly prosocline axial riblets which are finely granulose in the upper half of the whorls. Aperture elongate-ovoid; adapically angulated and probably with a very shallow siphonal canal. Parietal area convex and smooth lacking any lip; columellar lip forms a narrow and thin sheet. Its transition into the base is only recognisable by the abrupt onset of growth lines demarcating the callus. The terminal part of the columellar lip is slightly reflected forming a chink-like umbilicus.

*Remarks*: see genus.

*Distribution*: Only known from Thaytiniti.

***Pacaudiella flammulata*** **Harzhauser and Neubauer nov. gen. nov. sp.**

Figure 9h–i

*Holotype*: Fig. 9h, ONHM/TN/0032, height: 32 mm, diameter: 17 mm (TN3a).

*Paratype*: Fig. 9i, ONHM/TN/0033, height: 33 mm, diameter: 17 mm (TN3a).

*Stratum typicum*: Biomicritic limestones of the Zalumah Formation.

*Type locality*: Thaytiniti near Salalah, Sultanate of Oman.

*Age*: Priabonian (or early Rupelian).

*Name*: Referring to the colour pattern.

*Description*: Medium-sized, bulimoid shell of six teleoconch whorls with cyrtoconoid apex and high spire with weakly convex whorls; protoconch low dome-shaped but poorly preserved. Last whorl ovoid with slightly more pronounced convexity; a shallow groove appears below the granulose subsutural cord. Aperture largely destroyed; columella hollow, probably straight with delicate columellar swelling; parietal area smooth. Sculpture consisting of irregularly spaced prosocline growth lines, which are faintly granulose close to the suture of the last whorl. One of the specimens has a well-preserved colour pattern consisting of irregularly spaced, axially oriented, reddish-brown flammulae, becoming slightly broader towards the lower suture.

*Remarks*: Differs from *P. omanica* in its higher spire, the cyrtoconoid apex and the distinct groove below the subsutural cord. This species has the weakest sculpture of the achatinid-like species in the Omani Eocene. The colour pattern was already recognised by Pickford et al. (2014), documenting that the Omani Vidalilllidae developed a colour pattern convergent to that of some Achatinidae.

*Distribution*: Only known from Thaytiniti.

***Pacaudiella*** **cf.** ***flammulata*** **Harzhauser and Neubauer nov. gen. nov. sp.**

Figure 9j

*Material*: 1 (TN3a, ONHM/TN/0031).

*Measurements*: height: 29 mm, diameter: 15 mm

*Description*: Medium-sized, stout ovoid, bulimoid shell consisting of five low and moderately convex spire whorls and an ovoid, strongly convex last whorl. Protoconch low trochiform with pointed initial part and about one convex whorl of ca. 2.5 mm diameter; transition into teleoconch indistinct. Narrowly impressed suture adjoined by a prominent subsutural spiral cord. Shell surface smooth apart from weak growth lines (no microsculpture preserved or developed). Aperture ovoid, rather narrow, adapically angulated and with very shallow siphonal canal. Columella straight passing via a deep concavity into the convex parietal area. Inner lip narrow but slightly reflected forming a faint umbilical chink.

*Remarks*: This specimen differs from typical *P. flammulata* in its very stout ovoid shape and the pointed protoconch with a narrower initial part. It might represent simply a stout morph or a second species.

*Distribution*: Only known from Thaytiniti.

“Achatinoid clade” sensu Wade et al., 2006

Superfamily Streptaxoidea Gray, 1860

Family Streptaxidae Gray, 1860

**Genus** ***Gulella*** **Pfeiffer,** 1856

*Type species:* *Pupa menkeana* Pfeiffer, 1853; subsequent designation by Albers and Martens (1860). Recent, Eastern Africa.

***Gulella*** **nov. sp.**

Figure 10b

**Fig. 10:**
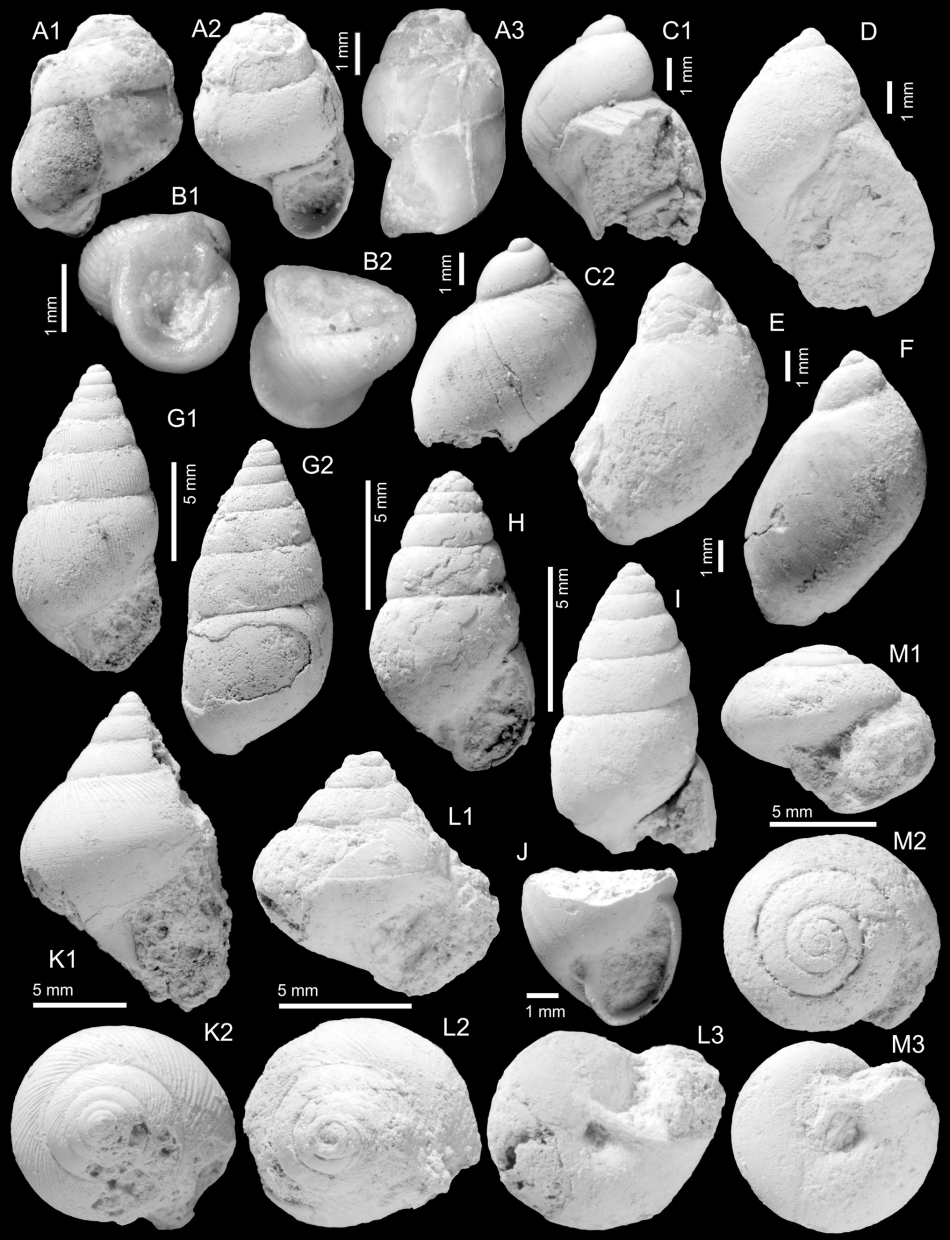
**a** *Goniodomulus solaniformis* Harzhauser and Neubauer nov. sp., Holotype, TN3a; ONHM/TN/0035. **b** *Gulella* nov. sp., HF1d, ONHM/HF/0010. **c**–**f** *Eoquickia omanensis* (Neubert and Van Damme, 2012), **c** TN3a, ONHM/TN/0036. **d** TN3a, ONHM/TN/0037. **e** TN3a, ONHM/TN/0038. **f** TN3a, ONHM/TN/0039. **g** *Cerastus praeinsularis* Neubert and Van Damme, 2012, HF1a, ONHM/HF/0011. **h**–**j** *Cerastus hyznyi* Harzhauser and Neubauer nov. sp., **h** Holotype, TN3c, ONHM/TN/0040. **i** Paratype, TN3c, ONHM/TN/0041. **j** Paratype, TN3c, ONHM/TN/0042. **k**–**l** *Sagdellina*? *arabica* (Neubert and Van Damme, 2012), **k** TN17b, ONHM/TN/0043., **l** TN17b, ONHM/TN/0044. **m** Helicarionoidea indet., TQ2; ONHM/TQ/0005

*Material*: 1 (HF1d, ONHM/HF/0010).

*Measurements*: aperture height: 2.1 mm, diameter: 2.4 mm.

*Description*: A single fragment comprising the last whorl and the aperture is available. Thus, no detailed information on total shell outline can be given. Last whorl convex, short with rapidly contracting base, covered by numerous, delicate strongly prosocline-sigmoidal axial ribs with slightly wider interspaces. Aperture sub-circular to weakly U-shaped; apertural margin formed by a flaring, very wide and reflected peristome with pustulose microsculpture; only the parietal lip is weaker and not well demarcated from the base. A prominent, elongate trigonal angular knob rests on the peristome and slightly protrudes to the base. It is accompanied by a deep palatal groove, becoming wider outwards but without forming a pronounced sinulus in the labrum.

*Remarks*: This is clearly a new species but we refrain from formally naming it due to the fragmentary preservation. The placement in the extremely polymorphic African genus *Gulella* is preliminary, but supported by Dai Herbert (pers. comm. 2014). Several extant species form comparable apertures with wide peristome and simple dentition, e.g. *Gulella infans* (Craven, 1880), which differs in the presence of a labral tooth. In addition, the Eocene occurrence fits well with the molecular phylogeny of the Streptaxidae published by Rowson et al. (2011), who discussed a major radiation of the group during the Palaeocene and Eocene including the appearance of *Gulella* sensu lato. Interestingly, Van Bruggen and Van Goethem (1997) considered an aperture type with no or simple dentition a plesiomorphic trait, which would also fit to the Omani species.

*Distribution*: Only known from Haluf.

**Genus** ***Goniodomulus*** **Harzhauser and Neubauer nov. gen.**

*Type species:* *Goniodomulus solaniformis* nov. sp.; Eocene, Priabonian (or early Rupelian), Sultanate of Oman.

*Diagnosis*: Small, ovoid shell of only three teleoconch whorls with very low protoconch. Teleoconch starting with a weakly convex whorl passing into strongly convex whorls with shallow subsutural depression on last whorl. Straight-opisthocline aperture; U-shaped with reflected peristome and detached parietal lip; umbilicus narrow.

*Included species*: Only the type species is known so far.

*Name*: Referring to the morphologically similar Mascarene *Goniodomus* Swainson, 1840 and the minute size.

*Remarks*: The extant *Goniodomus* Swainson, 1840 is similar in its ovoid outline, but is gigantic in relation to the tiny Eocene genus. Many extant streptaxids, such as *Gonaxis* Taylor, 1877 and allies, differ in their much larger size and strongly prosocline aperture. *Gulella* Pfeiffer, 1856 may develop a similar aperture without denticles and opisthocline orientation but is ovate-cylindrical with more whorls (see discussion on the genus in van Bruggen and van Goethem 1997).

*Gibbulinella* Wenz, 1920 is so far the only Palaeogene streptaxid genus known. Its type species *G. simplex* (Sandberger, 1872) from the Eocene of Italy which has a comparably simple aperture and a dome-shaped apex, but is cylindrical and it develops a thin parietal callus rather than a well-defined and detached lip. *Paracraticula* Oppenheim, 1890, which was listed as a streptaxid by Wenz (1923b), was placed in the Vertiginidae by Nordsieck (2014). Its Italian Eocene type species *P. umbra* (Oppenheim, 1890) has a strongly structured aperture and bulgy whorls, thus being completely different from the Omani species.

***Goniodomulus solaniformis*** **Harzhauser and Neubauer nov. gen. nov. sp.**

Figure 10a

*Holotype*: ONHM/TN/0035, height: 5.1 mm, diameter: 3.6 mm (TN3a).

*Stratum typicum*: Biomicritic limestones of the Zalumah Formation.

*Type locality*: Thaytiniti near Salalah, Sultanate of Oman.

*Age*: Priabonian (or early Rupelian).

*Name*: Referring to the potato-like shape (potato = *Solanum tuberosum*).

*Description*: Small, inflated-ovoid shell of three teleoconch whorls; protoconch very low and sunken in the high and weakly convex first teleoconch whorl (shell surface eroded, but the internal mould of the protoconch is preserved). The second whorl becomes more convex and develops a subsutural angulation. Last whorl strongly convex with weak suture; it lacks any angulation but develops a shallow subsutural concavity. Whorl height strongly increasing towards the U-shaped aperture. Apertural margin straight-opisthocline with continuous peristome; inner lip narrow, but distinctly reflected, passing into a wider basal lip; outer lip again narrowing; parietal lip swollen, slightly convex, and detached from base. No denticles or folds developed. Umbilicus narrow and circular; shell surface smooth.

*Remarks*: This species has very few teleoconch whorls. In respect to the well-developed aperture, however, we consider it to be a fully grown specimen.

*Distribution*: Only known from Thaytiniti.

“Non-achatinoid clade” sensu Wade et al., 2006

Unassigned “subclade” Elasmognatha Mörch, 1864

Superfamily Succineoidea Beck, 1837

Family Succineidae Beck, 1837

**Genus** ***Eoquickia*** **Harzhauser and Neubauer nov. gen.**

*Type species*: *Succinea omanensis* Neubert and Van Damme, 2012. Eocene, Priabonian (or early Rupelian), Sultanate of Oman.

*Diagnosis*: Very robust, medium-sized, ovate-attenuate Succineidae with pointed protoconch, moderately convex teleoconch whorls and high aperture; sculpture on last whorl consisting of prominent growth lines, which become very strong close to the suture, forming a weak crenulation in some specimens (see Fig. 10c1). Incised sutures adjoined by a weak subsutural swelling. Well-defined columellar plait and weakly thickened, concave columellar edge; umbilical chink developed only in subadult specimens.

*Included species:* Only the type species is known so far.

*Name*: Referring to the Eocene age and the extant Afro-Indian genus *Quickia* Odhner, 1950.

*Remarks*: The type species was originally placed with doubts in *Succinea* by Neubert and Van Damme (2012), who suggested that it might require the establishment of a new genus, a decision that was not possible at that time due to the poor preservation of the available material. Now, the combination of a very robust shell with weak subsutural swelling, prominent rib-like growth lines and well-defined columellar plait allows a separation from *Succinea* Draparnaud, 1801. Pickford et al. (2014) placed this species in *Quickia* Odhner, 1950, probably based on the Indo-African biogeography of the extant representatives of that genus. This view seemed to be supported by Patterson (1968), who considered *Quickia* a “primitive” succineid, based on anatomical traits. Extant *Quickia* species, such as *Quickia concisa* (Morelet, 1848), *Q. bensoni* (Pfeiffer, 1850), *Q. spurca* (Gould, 1851), and *Q. aldabraensis* Patterson, 1975, however, differ distinctly from the Omani species in their shorter and more rounded aperture and the deeply incised suture between base and aperture, which is accentuated by the strongly convex penultimate whorl.

The last comprehensive data compilation on fossil Succineidae goes back to Wenz (1923b). It is obvious from his (Europe-centred) synthesis that the family had two diversity maxima. The earlier radiation comprises species from the Palaeocene and Eocene of France and England followed by a very low diversity during Oligocene and early Miocene times. The second radiation started during the late Miocene and continues to the modern species, which did not evolve before Pliocene times. The species of this younger radiation represent typical *Succinea* and *Oxyloma* morphologies. The much older Palaeogene group, however, comprises morphologies with tiny spire and inflated last whorl (e.g. “*S*.” *boissyi* Deshayes, 1863; “*S*.” *brevispira* Deshayes, 1863, “*S*.” *palliolum* Sandberger, 1872). Wenz (1923b) placed these species in the extant genus *Brachyspira* Pfeiffer, 1856. This is a poorly known Caribbean genus (Patterson 1971) from which we conclude that a close relationship to the European Palaeogene species is unlikely. A second Palaeogene morphotype is represented by very high-spired shells with elongate aperture and slight basal angulation (e.g. “*S*.”. *sparnacensis* Deshayes, 1863; “*S*.” *headonensis* Wenz, 1919), which is also difficult to accommodate in modern genera as revised by Patterson (1971). Thus, we assume that the European Palaeocene–Eocene Succineidae belong to at least two extinct (unnamed) genera; the Eocene Omani species discussed here represents a third extinct genus.

***Eoquickia omanensis*** **(Neubert and Van Damme,** 2012 **)**

Figure 10c–f

*2012 *Succinea omanensis* Neubert and Van Damme: 12, Figs. 11–12.

2014 *Quickia omanensis*.—Pickford et al.: 95, 96.

*Material*: 130 (TN3a, illustrated specimens: ONHM/TN/0036–ONHM/TN/0039), 4 (TN8), 142 (TN12).

*Measurements*: Largest specimen: height: 9.5 mm, diameter: 5.9 mm.

*Description*: Medium-sized and solid Succineidae of ovate-attenuate outline with pointed protoconch with a slightly immersed initial part. The teleoconch whorls are moderately convex, gain rapidly in width with an increasingly oblique suture; last whorl high, weakly convex and with strong growth lines, which are most prominent along the upper suture. There, the last whorl develops a narrow subsutural bulge, which may be somewhat crenulated by the growth lines. Despite that structure, fully grown shells have a rather continuous conical spire outline. Columellar edge weakly concave and slightly thickened, passing into a well-defined columellar plait on the base (thick in subadult individuals, but thin in fully grown specimens) and a thin, strongly convex basal lip. A narrow umbilical chink is developed in subadult shells; apertural margin prosocline.

*Distribution*: Only known from Thaytiniti and the Haluf region.

Unassigned “subclade”

Superfamily Clausilioidea Gray, 1855

Family Clausiliidae Gray, 1855 (text by H. Nordsieck)

*Remarks*: The clausiliid fauna from the Zalumah Formation of Taqah consists of three species, two of a genus of the subfamily Eualopiinae, and one of a genus of the subfamily Laminiferinae. All species and genera are new to science. All specimens are internal moulds, so that only part of the characters are accessible for examination. Therefore and because the material is scarce, the information on the species given in this contribution has to be treated with caution. It is of special interest that at least two species have oospiric shells (shell egg- or pupa-shaped with blunt apical part and high D/H value; see Nordsieck 2015). This points to an age of the fauna not younger than Eocene, because in Oligocene or Neogene faunas (at least in Europe) no oospiric clausiliids have been found.

Abbreviations: *D* = width; *D*_A_ = aperture width; *H* = height; *H*_A_ = aperture height; *R*_1_ = rib number per 1 mm of a whorl; *W* = whorl number.

Subfamily Eualopiinae H. Nordsieck, 1978

Tribe Rillyini H. Nordsieck, 1985

*Remark*: Rillyini are characterized by a shell with few whorls and a non-apostrophic aperture with adnate or interrupted peristome; lunellar of lunella type (Nordsieck 2015).

**Genus** ***Omanillya*** **H. Nordsieck nov. gen.**

*Type species:* *Omanillya lunellifera* nov. sp. Eocene, Priabonian (or early Rupelian), Sultanate of Oman.

*Diagnosis*: Shell oviform, oospiric; cervix rounded; peristome interrupted; lunellar consisting of principal plica and lunella or only principal plica.

*Name*: Name derived from Oman and *Rillya*, because of its similarity with *Rillya* Munier-Chalmas in Fischer, 1883 and related genera.

*Remarks*: The new genus is placed within the Eualopiinae, tribe Rillyini, because of its interrupted peristome and the presence of a lunella. The relative shell width (D/H) and the relative aperture height (H_A_/H) are like those of *Neniopsis* Wenz, 1920, but the shell is oviform. Contrary to *Neniopsis*, but as in *Pararillya* H. Nordsieck, 2002, palatal plicae are present, in the type species even a lunella is evident. Therefore, it is possible that the shell was also furnished with a clausilium.

***Omanillya lunellifera*** **H. Nordsieck nov. gen. nov. sp.**

Figure 11a–b

**Fig. 11:**
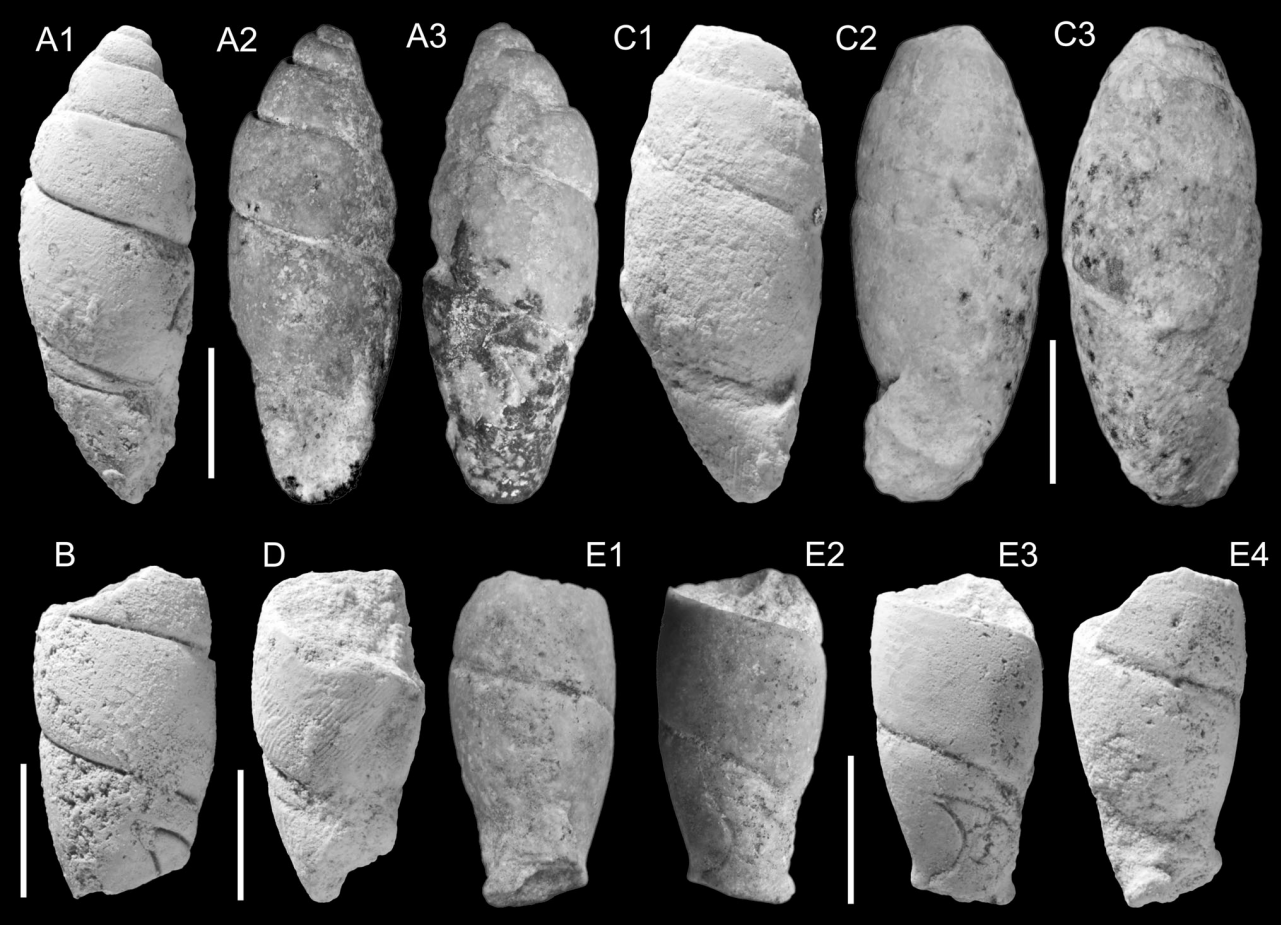
Clausiliidae. **a**–**b** *Omanillya lunellifera* H. Nordsieck nov. sp. **a** Holotype, TQ3; ONHM/TQ/0006., **b** Paratype, TQ3; ONHM/TQ/0007, **c**–**d** *Omanillya costellata* H. Nordsieck nov. sp. **c** Holotype, TQ13-2; ONHM/TQ/0008., **d** Paratype, TQ13-2; ONHM/TQ/0009., **e** *Omanifera euclista* H. Nordsieck nov. sp., Holotype, TQ3; ONHM/TQ/0010. *Scale bar* 5 mm

*Material*: holotype (complete shell) (TQ3, ONHM/TQ/0006); 1 paratype (fragment with aperture) (TQ3, ONHM/TQ/0007).

*Measurements*: holotype (Fig. 11a): H 18.9 mm, D 6.6 mm, H_A_ (inner margin) 5.2 mm, D_A_ 4.2 mm; W . Paratype (Fig. 11b): H (fragment) 11.5 mm, D 6.8 mm; W 2½. R_1_ (before penultimate whorl, extrapolated): 8, (body whorl) 7.

*Stratum typicum*: Biomicritic limestones of the Zalumah Formation.

*Type locality*: Taqah, near Salalah, Sultanate of Oman.

*Age*: Priabonian (or early Rupelian).

*Name*: Latin lunellifera = bearing a lunella.

*Description*: Shell slender-oviform, whorls somewhat rounded; rib-striated; cervix rounded; aperture rhombic, rounded below, peristome (only inner or outer upper end preserved) interrupted, with straight inner margin; lunellar dorsally situated, principal plica ending about dorsally or dorsal-dorsolaterally, in front converging with the suture; upper palatal plica diverging from inner end of principal plica, continuous with lunella by an arch, lunella (in holotype) steep, traceable only to the middle of the body whorl.

*Remarks*: In the aperture in one specimen a trace of inferior lamella is recognizable.

*Distribution*: Only known from Taqah.

***Omanillya costellata*** **H. Nordsieck nov. gen. nov. sp.**

Figure 11c–d

*Material*: holotype (nearly complete shell without apex) (Fig. 11c, TQ2, ONHM/TQ/0008); 1 paratype (fragment with aperture), (Fig. 11d, TQ2, ONHM/TQ/0009).

*Measurements*: holotype: H (without apex) 17.9 mm, actual height estimated at ~19 mm, D 7.5 mm, H_A_ (outer margin) 5.0 mm, D_A_ ~ 4.5 mm; W 3½. paratype: H (fragment) 13.0 mm, D 6.9 mm, H_A_ ~ 5.1 mm, D_A_ ~ 4.5 mm, W 1½. R_1_ (penultimate whorl): 6½, 7, 6, (body whorl) 5, 5.

*Stratum typicum*: Biomicritic limestones of the Zalumah Formation.

*Type locality*: Taqah, near Salalah, Sultanate of Oman.

*Age*: Priabonian (or early Rupelian).

*Name*: Latin costellata = rib-striated.

*Description*: Shell more ventricose than in the preceding species, whorls less rounded; distinctly rib-striated (additionally fine radial striae present); cervix rounded, body whorl somewhat expanded before aperture; peristome (only outer upper end preserved) interrupted; lunellar dorsally (?) situated, principal plica ending about dorsally or nearly dorsolaterally, in front less converging with the suture; no lunella recognizable.

*Distribution*: Only known from Taqah.

Subfamily Laminiferinae Wenz, 1923

*Remark*: In the Laminiferinae are assembled all European clausiliids with apostrophic aperture. All apostrophic clausiliids have a lunellar of lunella type (Nordsieck 2007: 12–13). The new genus is not affiliated to any particular tribe.

**Genus** ***Omanifera*** **H. Nordsieck nov. gen.**

*Type species*: *Omanifera euclista* nov. sp. Eocene, Priabonian (or early Rupelian), Sultanate of Oman.

*Diagnosis*: Shell with body whorl much descending before aperture, (probably) apostrophic; cervix with basal and dorsal elevations; aperture protruding; lunellar consisting of principal plica, lunella and posterior lower palatal plica.

*Name*: Name derived from Oman and *Laminifera*, because of its similarity to *Laminifera* O. Boettger, 1863.

*Remarks*: Because of the descending body whorl the shell is judged to be apostrophic; therefore, the new species is placed within the Laminiferinae. The shell exhibits a complete lunellar as in *Laminifera* and was probably furnished with a clausilium, but it is larger and more ventricose than that of *Laminifera*. Because only a fragment is available, it cannot be determined whether the shell is oospiric or not.

***Omanifera euclista*** **H. Nordsieck nov. gen. nov. sp.**

Figure 11e

*Material*: holotype (fragment with aperture) (TQ3, ONHM/TQ/0010).

*Measurements*: holotype: H (fragment) 12.0 mm, D 6.0 mm, H_A_ not measurable, D_A_ 3.8 mm, W 2. R_1_ (body whorl, extrapolated) 8.

*Stratum typicum*: Biomicritic limestones of the Zalumah Formation.

*Type locality*: Taqah, near Salalah, Sultanate of Oman.

*Age*: Priabonian (or early Rupelian).

*Name*: Greek euclista = with well-developed closing apparatus.

*Description*: Shell more slender than in the *Omanillya* species; whorls less rounded, body whorl narrowed, much descending before aperture; basal keel and dorsal elevation of cervix recognizable; aperture (outer upper end preserved) protruding; lunellar about dorsally situated, principal plica ending dorsal-dorsolaterally, in front converging with the suture and descending; upper palatal plica very short, connected with lunella, upper part of lunella oblique, continuous with the steep lower part by an obtuse angle, posterior lower palatal plica present, somewhat bent, connected with lunella by almost a right angle, ending on the palatal wall between basal keel and dorsal elevation.

*Distribution*: Only known from Taqah.

Limacoidea incertae sedis

Superfamily Helicarionoidea Bourguignat, 1877

**Genus** ***Sagdellina*** **Cossmann,** 1889

*Type species:* *Helix Chevallieri* Cossmann, 1886; original designation. Eocene, France.

***Sagdellina*** **?** ***arabica*** **(Neubert and Van Damme,** 2012 **)**

Figure 10k–l

*2012 *Trochozonites arabica* Neubert and Van Damme: 20, Fig. 25.

2014 *Edouardia arabica*.—Pickford et al.: 95.

*Material*: 4 specimens (TN17b, illustrated specimens: ONHM/TN/0043–ONHM/TN/0044).

*Measurements*: Largest specimen: height: 17 mm, diameter: 12.5 mm.

*Description*: Small and robust turbiniform shell with six whorls; conical spire with weakly convex whorls and an apical angle of 65°–75°. Sutures impressed, adjoined by a weak subsutural cord. Spire fragments or subadult shells display a strong keel along the periphery, hidden by the proceeding whorl in adult specimens. Last whorl strongly allometric, rapidly widening and more convex than the spire whorls. Periphery well rounded without keel or angulation. Strongly prosocline-convex to orthocline-convex, densely spaced, fold-like axial ribs, fading out towards the lower suture; with narrow and smooth, adapically widening interspaces. Additional axial ribs may appear in the interspaces, resulting in a pseudo-bifurcate pattern. Weak and wavy spiral grooves form the spiral sculpture; these grooves are best developed along the periphery of the last whorl and on the base, but are faint in the upper half of the whorls. Base convex with distinct growth lines and moderately wide and deep umbilicus. Aperture largely destroyed; inner lip straight and reflected, passing into a simple and thin basal lip; parietal area smooth.

*Remarks*: When introducing this species, Neubert and Van Damme (2012) had only spire fragments at hand. Therefore, they were not aware of the allometric growth of last whorl, which lacks the angulation and keel at the periphery typical of the spire whorls. This induced these authors to place the taxon in the Urocyclidae genus *Trochozonites* Pfeffer, 1883, which clearly differs in its low and weakly convex base and the generally smaller shells. Pickford et al. (2014) proposed a placement of this species in the Cerastidae genus *Edouardia* Gude, 1914. The robust shell, the subsutural swelling, the spiral grooves and the mode of axial rib formation of the Eocene fossil contradict a close relation with *Edouardia* and its allies.

Among the Eocene circum-Tethyan fauna only *Sagdellina* Cossmann, 1889 is reminiscent of the Omani species. The specimens described by Cossmann (1889) are all smaller and the generic placement of the Omani species in *Sagdellina* may be doubted. A distinctly larger specimen of an undescribed *Sagdellina* species from the Eocene of Rilly (France), stored in the collection of the Senckenberg Museum, however, suggests that this genus also contains larger species.

*Distribution*: Only known from Thaytiniti and the Haluf area.

**Helicarionoidea indet.**

Figure 10m

*Material*: 1 specimen (TQ2, ONHM/TQ/0005).

*Measurements*: Height: 8 mm, diameter: 10 mm.

*Description*: Only a single internal mould is available, which shows a stout helicoid shell comprising 4.5 whorls. The low, dome-shaped spire consists of convex spire whorls with incised sutures (as far as this can be judged from an internal mould); the last whorl has a weak subsutural angulation followed by a weakly convex flank and a slightly angulated periphery, which is situated in the lower 2/5 of the last whorl; base evenly convex. The terminal part of the last whorl grows slightly downwards; consequently, the suture is shifting slightly below the periphery towards the aperture. Aperture destroyed, but based on the terminal parts of the last whorls at least the basal part was slightly flaring (note that the peripheral part of the aperture is broken off, causing a misleading impression of a rather narrow aperture). Umbilicus of the mould wide and funnel-shaped, but it remains unclear whether the umbilicus was open or concealed by a callus. No denticles or other structures are developed in the inner part of the aperture.

*Remarks*: The identification of the specimen is highly tentative due to the poor preservation and we place this species in the Helicarionoidea based mainly on the overall shape and aperture. The specimen is also reminiscent of some *Tayloria* species (e.g. *T. jouberti* Bourguignat, 1889, *T. striata* Verdcourt, 1958), but the mainly Middle African distribution of this genus (Schütt et al. 1991) casts doubt upon an occurrence in the Arabian Eocene. Moreover, the angulated basal part of the aperture would be atypical for *Tayloria*.

*Distribution*: Only known from Taqah.

Informal group Orthurethra Pilsbry, 1900

Superfamily Enoidea Woodward, 1903

Family Cerastidae Wenz, 1923

**Genus** ***Cerastus*** *Albers,* 1860

*Type species:* *Bulimus distans* Pfeiffer, 1856. Recent, Persian Gulf.

***Cerastus praeinsularis*** **Neubert and Van Damme,** 2012

Figure 10g

*2012 *Cerastus praeinsularis* Neubert and Van Damme: 18, Figs. 18–19.

2014 *Pseudoglessula*.—Pickford et al.: 95, Fig. 3d.

*Material*: 5 (HF1a, ONHM/HF/0011), 12 (HF1d).

*Measurements*: Largest specimen: height: 15 mm, diameter: 7.5 mm (HF1d).

*Description*: Pupoid shell of 7–8 whorls; protoconch comprising about 1.5 strongly convex, probably slightly granulose whorls. Onset of teleoconch marked by distinct, densely spaced, rounded, slightly prosocline-convex axial ribs, which continue on the entire teleoconch. First three teleoconch whorls elongate-turreted but strongly convex, followed by distinctly wider and higher whorls, resulting in a coeloconoid spire outline; sutures narrowly incised. Aperture moderately wide, adapically angulated, basally convex with straight columella, which passes via an angulation into the weakly convex parietal area. Inner lip thin and reflected partly covering the broad umbilicus. Umbilicus delimited by a broad umbilical crest.

*Remarks*: The holotype of this species is a poorly preserved specimen (Neubert and Van Damme 2012), which lacked the very distinct axial ribbing and large parts of the aperture. These authors based their generic placement on the assumed antiquity of the genus and the similarities in umbilical features especially with Soqotran cerastids. In addition, the densely spaced, rounded axial ribs are typical of some of these species as described by Neubert (2005), but also of some African species such as *Cerastus moellendorffi* Kobelt, 1901. We are not aware of any comparable Palaeogene species. The Eocene *Procerastus vicentina* (Oppenheim, 1890) is stout ovoid. The earliest record of a comparable fossil cerastid comes from the lower Miocene of Kenya described as *Cerastus* cf. *moellendorffi* Kobelt, 1901 by Newton (1914). *Cerastus miocenicus* Verdcourt, 1963, from the Miocene of Kenya, has a more ovoid outline and a broader spire.

A rather slender, subadult specimen of this species from Taqah was illustrated by Pickford et al. (2014) and identified as *Pseudoglessula* (being indeed reminiscent of some species, such as *P. umbilicata* Pilsbry, 1919). The African subulinid genus *Pseudoglessula* Boettger, 1892, however, differs in its more slender elongate outline, the truncate columella and the very characteristic, strongly sculptured protoconch (Pilsbry 1919).

*Distribution*: Known from the Haluf region and Taqah.

***Cerastus hyznyi*** **Harzhauser and Neubauer nov. sp.**

Figure 10h–j

*Holotype*: Fig. 10h, ONHM/TN/0040, height: 11 mm, diameter: 5.9 mm (TN3c).

*Paratype*: Fig. 10i, ONHM/TN/0041, height: 10 mm, diameter: 5 mm (TN3c).

*Paratype*: Fig. 10j, ONHM/TN/0042, diameter: 4.8 mm (TN3c).

*Additional material:* 105 (TN3a), 10 (TN3c), 1 (TN8).

*Stratum typicum*: Biomicritic limestones of the Zalumah Formation.

*Type locality*: Thaytiniti near Salalah, Sultanate of Oman.

*Age*: Priabonian (or early Rupelian).

*Name*: In honour of Matúš Hyžný, palaeontologist and crustacean specialist.

*Description*: Small, pupoid shells of six teleoconch whorls; obtuse protoconch consisting of ca. 1.5 low and convex whorls. Spire whorls convex and bulging, separated by very narrow and deeply incised sutures. Sculpture ranging from smooth to weakly ribbed with densely spaced, low and slightly prosocline axial riblets. Ovoid, moderately wide aperture with adapical angulation and weakly concave columella, passing via an angulation into the nearly straight parietal area. Inner lip thin and reflected, covering large parts of the narrow umbilicus. A distinct crest accompanies the umbilicus. Outer lip thin, not flaring.

*Remarks*: This species differs from *Cerastus praeinsularis* Neubert and Van Damme, 2012 by its lesser dimensions, the distinctly lower last whorl, the strongly convex spire whorls and the narrower umbilicus. Moreover, its sculpture is much finer. *Cerastus pseudoena* Neubert and Van Damme, 2012, which was also described from the Eocene of Oman, differs in the less bulging whorls and slightly coeloconoid spire outline.

*Distribution*: Only known from Thaytiniti.

## Discussion and conclusions

The focus of this study is the taxonomy and systematics of the gastropod species from the Zalumah Formation, Oman. No new data on the lithology and facies of the sections is added herein and the biostratigraphic value of the gastropod assemblages is rather low at this stage due to the fully endemic character of the fauna and the lack of comparable Afro-Arabian Palaeogene assemblages. Nevertheless, the gastropod faunas allow some palaeoecological conclusions to be drawn at the local scale and are highly significant in a palaeobiogeographical context.

*Palaeobiogeography*: Eocene continental mollusc faunas of the Tethys Region and Europe are still poorly understood. The most diverse assemblages are known from the Paris Basin and Southern France, the Hampshire Basin in England, the Upper Rhine Graben in Germany, the Veneto region in Italy and the Fayum Basin in Egypt. All these assemblages have received little attention since the syntheses of Wenz (1923–1930, 1942), although Jodot (1938, 1953a, b, 1957a, b), Abbas (1962, 1967), Plaziat (1973), Szöts (1953), Esu (1984) and Kadolsky (2015) added some information concerning northwestern African, Egyptian, French, Hungarian, Balearic, and German faunas. Aside from the well-preserved French faunas, most others are taphonomically strongly biased and often only deformed shells or casts and moulds are available. Moreover, the few more or less well-described assemblages derive from different horizons covering only parts of the ca. 22 million years of Eocene time and are mostly older than the Priabonian Omani fauna. Thus, these spotty data yield only a vague impression of circum-Tethyan Eocene continental biogeography.

In any case, the Omani assemblages contain not a single species known from any other region. This might point to the presence of a distinct biogeographic province during the late Eocene of Arabia or may just be a reflection of the extremely sparse non-marine Eocene fossil record in the Tethys region. At the genus and family level, some clear relations may be proposed: in the Hydrobiidae?, *Salalahia* is closely related to *Nystia* from the lower Rupelian of western Europe; in the Pomatiidae, *Omanitopsis* is closely related to *Bembridgia* from the Priabonian of France and England; *Procyclotopsis* and *Palaeocyclotus* occur in the Tethyan island faunas of the Italian Veneto; the Ampullariidae of Oman are related to those of Egypt, which was part of the southern Tethys coast. In contrast, an example for a certain biogeographic distinctiveness of the Omani faunas is represented by the diverse Vidaliellidae. This family is widespread across the Tethys Region and developed a second centre of diversity in northwestern Africa, with radiation of the genus *Vicentinia* as described by Jodot (1953a, 1957b). Interestingly, the Vidaliellidae did not reach northern Europe. These faunistic relations to the European and African faunas point to an Eocene (Priabonian) age supporting previous proposals. A Rupelian age, however, cannot be definitely excluded.

As already proposed by Neubert and Van Damme (2012) and Pickford et al. (2014) some of the taxa might represent ancestors of extant African and Arabic taxa. Among these, the occurrences of *Lanistes, Pila* and *Gulella* s.l. are rather reliable and are most probably not just a result of convergences. Especially *Gulella* fits also to expected radiations based on molecular data (Rowson et al. 2011). In addition, the Pomatiidae, herein united in the new genus *Omanitopsis*, might be closely related to extant taxa such as *Cyclotopsis*, as discussed also by Neubert and Van Damme (2012). East Africa is a centre of diversity of modern Pomatiidae, which suggests a long-standing presence of this group in the region, and this is supported by the presence of four species in the Zalumah Formation. Other groups, such as the already mentioned Vidaliellidae and some of the frequent hydrobioids (*Arabiella*, *Pyrgulella)* have no modern counterparts.

*Palaeoecology* Neubert and Van Damme (2012) already interpreted the depositional environment as “extensive freshwater swamps rather than lakes or rivers” and this interpretation is fully confirmed by our data. Neubert and Van Damme (2012) also postulated a “marked seasonal difference in dry and rainy season”, which is supported by the internal structures of the clausiliids, which display adaptations to periodic aridity.

Aquatic genera bound to freshwater settings, such as *Planorbarius, Lanistes* and *Pila,* are abundant at Thaytiniti. Large numbers of hydrobiids and succineids appear in samples TN3a and TN12 suggesting the presence of extended wetlands. The Ampullariidae are most common in samples TN8 and TN15b (*Lanistes*) and TN3a and TN3c (*Pila*). Extant *Pila* species occupy a broad range of freshwater habitats such as temporary pools, ponds, swamps, and stony beaches and even small streams (Brown 1994). Overall, however, *Pila* prefers lentic conditions (Sreejith 2014). Similarly, *Lanistes* may be found in standing and flowing freshwater systems (Brown 1994). The mass occurrence of the hydrobioid *Salalahia thaytinitiensis* in sample Thaytiniti 3a may indicate slightly increased salinity based on the interpreted relationship with *Nystia*. Elsewhere any marine-brackish influence is unlikely based on the obligate freshwater dwellers Ampullariidae and Planorbidae. Moreover, none of the brackish-marine potamidid, batillariid, and cerithiid genera, which inhabited the Palaeogene coastal swamps of the southern Tethys (e.g., Harzhauser 2007; Harzhauser et al. 2013) are detected in the Zalumah Formation.

At Haluf, terrestrial species of the Pomatiidae predominate the samples along with rare *Gulella* and *Cerastus*.

Only a few species occur at more than one locality and the faunistic overlap is limited to the samples TN17b at Thaytiniti and HF1d at Haluf. This pattern might suggest different ecological conditions and/or stratigraphic differences. The relative position of Taqah to these two sections cannot be resolved based on the mollusc fauna.

In conclusion, the new samples from the Eocene of the Salalah area comprise 27 gastropod species (Table 3). Of these, 15 species are new to science and four species are left in open nomenclature but clearly represent undescribed species. Only eight of these species were previously described by Neubert and Van Damme (2012). In addition, several fragments of subulinid-like species and subadult Vidaliellidae are not considered herein due to the poor preservation, but they document the presence of additional undescribed species. Hence, the Eocene continental mollusc fauna of Arabia is still under-sampled and new campaigns are likely to add new information.

**Table 3:** Distribution of the discussed taxa in the Zalumah Formation at the localities Taqah, Thaytiniti, and Haluf (numbers indicate available specimens)

Species	Taqah		Thaytiniti									Haluf				
	TQ2	TQ3	TN3a	TN3b	TN3c	TN8	TN9	TN12	TN15b	TN15c	TN17b	HF1a	HF1d	HF3b	HF4b	HF4c
*Pila neuberti* Harzhauser and Neubauer nov. sp.			15	1	7		2	8								
*Pila* sp.				1												
*Lanistes tricarinatus* (Neubert and van Damme, 2012)			1			30			9						2	
*Carnevalea thaytinitiensis* (Neubert and van Damme, 2012)						179			5							
*Salalahia thaytinitiensis* Kadolsky, Harzhauser and Neubauer nov. sp.			710		15			20								
*Arabiella arabica* Kadolsky, Harzhauser and Neubauer nov. sp.											22		2			
*Pyrgulella parva* Harzhauser, Neubauer and Kadolsky nov. sp.			1													
*Omanitopsis praecursor* (Neubert and van Damme, 2012)			412			164	129	429	5		355					
*Omanitopsis vandammei* Harzhauser and Neubauer nov. sp.											43	64		28	129	99
*Procyclotopsis eocenica* Harzhauser and Neubauer nov. sp.													43		6	9
*Palaeocyclotus kueschelmi* Harzhauser and Neubauer nov. sp.	142	27														
*Planorbarius* sp.			3		3		1				3					
*Arabicolaria omanensis* (Neubert and van Damme, 2012)			4							1						
*Arabicolaria arabica* Harzhauser and Neubauer nov. sp.			5							2						
*Arabicolaria sculpturata* (Neubert and van Damme, 2012)	4															
*Pacaudiella omanica* Harzhauser and Neubauer nov. sp.										1						
*Pacaudiella flammulata* Harzhauser and Neubauer nov. sp.			2													
*Pacaudiella* cf. *flammulata* Harzhauser and Neubauer nov. sp.			1													
*Gulella* nov. sp.													1			
*Goniodomulus solaniformis* Harzhauser and Neubauer nov. sp.			1													
*Eoquickia omanensis* (Neubert and van Damme, 2012)			130			4		142								
*Omanillya lunellifera* Nordsieck nov. sp.		2														
*Omanillya costellata* Nordsieck nov. sp.	2															
*Omanifera euclista* Nordsieck nov. sp.		1														
*Sagdellina*? *arabica* (Neubert and van Damme, 2012)											4					
Helicarionoidea indet.	2															
*Cerastus praeinsularis* Neubert and van Damme, 2012												5	12			
*Cerastus hyznyi* Harzhauser and Neubauer nov. sp.			105		10	1										
